# Nanoscale Diamond-Based Formulation as an Immunomodulator and Potential Therapeutic for Lymphoma

**DOI:** 10.3389/fphar.2022.852065

**Published:** 2022-04-04

**Authors:** Ankush Paladhi, Abhinandan Rej, Debanjan Sarkar, Ranjeet Singh, Sankar Bhattacharyya, Prasanta Kumar Sarkar, Pulak Kanti Kar, Partha Pratim Manna, Sumit Kumar Hira

**Affiliations:** ^1^ Cellular Immunology Laboratory, Department of Zoology, The University of Burdwan, Burdwan, India; ^2^ Immunobiology Lab, Department of Zoology, Sidho Kanho Birsha University, Purulia, India; ^3^ Immunobiology Laboratory, Department of Zoology, Institute of Science, Banaras Hindu University, Varanasi, India; ^4^ Department of Rasashastra, J. B. Roy State Ayurvedic Medical College and Hospital, Kolkata, India; ^5^ Department of Panchakarma, J. B. Roy State Ayurvedic Medical College and Hospital, Kolkata, India

**Keywords:** Heerak Bhasma, ayurveda, nanodiamond, lymphoma, dendritic cell, T cell

## Abstract

Integrative medicine practices, such as Ayurveda, are popular in India and many South Asian countries, yet basic research to investigate the concepts, procedures, and medical benefits of ayurvedic products has received little attention and is not fully understood. Here, we report a functional nanodiamond-based traditional Ayurvedic herbomineral formulation, *Heerak Bhasma* (Ayu_ND), for the treatment of solid tumors called Dalton’s lymphoma generated in CD1 mice. Ayu_ND-mediated immunostimulation significantly reduces tumor cell proliferation and induces apoptosis aided by the active participation of dendritic cells. Immunomodulatory Ayu_ND treatment is highly immunostimulatory and drives dendritic cells to produce TNF-α. Treatment with Ayu_ND significantly reduces the tumor volume, inhibits metastasis in distant vascularized organs, and increases the life span of tumor-bearing animals compared with untreated littermates. These events were associated with elevated serum levels of the protective cytokines IFN-γ and TNF-α and downregulated the disease, exacerbating TGF-β. Ayu_ND-mediated therapeutic success was also accompanied by the depletion of regulatory T cells and enhanced vaccine-induced T-cell immunity, guided by the restoration of the memory CD8^+^ T-cell pool and prevention of PD-1-mediated T cell exhaustion*.* The results provide a basis for further evaluation of ayurvedic formulations and drug efficacy in treating cancers.

## Introduction

Nanodiamonds, due to their specific physicochemical properties (e.g., unique sizes and adaptable surface functionalizing modifications), are currently under large-scale investigation for their potential utilization as drug delivery agents for chemotherapeutic drugs. In addition, nanodiamonds are also used as devices for molecular imaging and grading of tumors or even as therapeutic assessments of tumors (Yang et al., 2010). Nanodiamond (ND) has emerged as a promising candidate owing to its biocompatibility, extensible synthetic methods, and unique surface-mediated binding of a large variety of bioactive molecules. These properties make ND unique among many other alternatives, including polymeric micelles, ceramic nanoparticles, viral capsid-derived nanoparticles, and silica nanoparticles. Diamond nanoparticles or nanodiamonds are single crystals of diamond consisting of carbon as the basic component with high physical and chemical properties (Chauhan et al., 2020). The amazing properties of NDs have attracted researchers’ interest in various fields of applications, including components of biosensors and enhancement of imaging efficiency (Mohan et al., 2010; Hsu et al., 2014), targeted drug delivery and sustained drug release (Kossovsky et al., 1995; Huang et al., 2007; Purtov et al., 2010; Chow et al., 2011; Madamsetty et al., 2019) to boost therapeutic efficacy, and improved safety profiles. NDs are also used as biocompatible composites and implants (Mochalin et al., 2012) and can provide stable solid supports for the synthesis of peptides (Bondar et al., 2004; Huang et al., 2004; Moore et al., 2012; Zhang et al., 2014). Nanodiamonds are also used as targeted drug delivery vehicles for bone diseases and bone regeneration (Krueger, 2008; Zhang et al., 2011; Okamoto, 2013).

Despite its diversified applications in health science, the influence of various nanoparticles on the immune system is poorly understood. It was recently shown that exposure to a mixture of nanodiamond and nanoplatinum (NP)-coated material activates murine T cells (Ghoneum et al., 2010b). Recent studies have shown that the ND/NP combination activates human dendritic cells (DCs) and DC-driven CD4^+^ naïve T-cell proliferation *in vitro*, which may be useful in boosting immune responses in cancer treatment (Ghoneum et al., 2010a). Dendritic cells play a significant role in boosting host immune defense, such as removal of foreign pathogens and suppression of tumorigenesis.

The use of diamond nanoparticles in modern medicine can be traced back to their use in traditional ancient Indian Ayurvedic medicine for healing cancer by boosting the immune system (Kulkarni, 2010; Sarkar and Chaudhary, 2010; Sharma, 2014). Ayurvedic preparations typically consist of a mixture of herbal- and animal-derived products, minerals and other metals (Mukherjee and Wahile, 2006; Sarkar and Chaudhary, 2010; Chaudhary, 2011; Pal et al., 2014). Diamond-derived ayurvedic medicine is called *Heerak Bhasma* (incinerated diamond powder) (Kulkarni, 2010; Sharma, 2014; Sumersingh Rajput et al., 2016). Several studies have focused on the effect of ayurvedic bhasma on human health, thus supporting the notion that these bhasmas may have a specific impact on the host immune system implicated in disease initiation and progression (Verma and Prasad, 1995; Singh et al., 2010; Paul and Sharma, 2011; Umrani and Paknikar, 2011; Jagtap et al., 2013; Umrani et al., 2013; Chaudhari et al., 2016; Kale and Rajurkar, 2018; Kantak et al., 2019). However, no experimental evidence has been reported regarding the physiological and immunological effects of *Heerak Bhasma* (*Ayu_ND*). Integrative medicine practices, such as Ayurveda, are popular in India and many South Asian countries, yet basic research to investigate the concepts, procedures, and medical benefits of Ayurvedic products has received little attention and is not fully understood.

The present work aimed to prepare *Heerak Bhasma* (*Ayu_ND*) according to the procedure mentioned in the ancient Indian text *Rasaratna Samuccaya* (Supplementary Figure S1) (Kulkarni, 2010) and test their localization, entry and impact (direct antitumor activity as well as impact on physiological and immunological parameters) on mammalian cells compared with similar treatment with commercially synthesized NDs (C_ND). In the present study, the as-prepared Ayu_ND was analyzed for quality and other parameters described in ayurvedic texts along with modern technologies such as transmission electron microscopy (TEM), scanning electron microscopy (SEM), energy dispersive X-ray analysis (EDX), X-ray diffraction (XRD), Fourier transform infrared spectroscopy (FTIR), and dynamic light scattering (DLS). to determine the nature and form of the prepared material (bhasma). Ayu_ND treatment of murine spleen-derived DCs resulted in *1*) increased expression of costimulatory molecules CD40 and CD86 on DCs, *2*) upregulation of the levels of DC-derived cytokines such as TNF-α and IFN-γ, and *3*) increased antigen-specific proliferation of CD4^+^ and CD8^+^ T cells. In addition, Ayu_ND augmented DC-mediated antilymphoma activity *in vitro* and *in vivo*. Oral therapy with Ayu_ND likely stimulates dendritic cells *in vivo* and initiates a series of tumoricidal immune functions, including the effector memory response mediated by the CD8 arm of T-cell immunity. ND therapy severely restricts the regulatory T-cell network and limits the synthesis of disease-exacerbating TGF-β while augmenting the production of disease-fighting cytokines such as TNF-α and IFN-γ. Altogether, our data demonstrated a novel therapeutic opportunity for traditional Indian medicine and its relevance to fight against dreaded diseases such as lymphoma with detailed analysis of immune surveillance mechanisms.

## Material and Methods

### Ethics Statement

This study was carried out in strict compliance with the recommendations for CPSEA, MoEF, and GOI. The protocol was approved by the Institutional Dissection Monitoring Committee, The University of Burdwan. Mice were euthanized by fulfilling the ARRIVE guidelines.

### Reagents and Antibodies

Commercially prepared nanodiamond powder (C_ND) was purchased from Millipore Sigma, USA (Cat No. 636444). The raw diamond powder was collected from Diamond Market, Surat, India. Primary or conjugated anti-mouse CD40, CD86, CD3, CD4, CD8, CD11c and CD11b and CD25, TNF-α, TGF-β, and Foxp3 were purchased from BioLegend USA. Information about the antibodies is summarized in Supplementary Table S1 included in the supplementary information file. β-actin was purchased from Cell Signaling Technology. Annexin-FITC apoptosis assay kits and ELISA kits were procured from BioLegend USA. LPS and all other fine chemicals were purchased from Millipore Sigma, USA.

### Preparation of Ayu_ND and Characterization

Ayu_ND (Heeraka Bhasma) was prepared by following the purification and incineration methods described in the texts of Ayurveda (Supplementary Figure S1) (Kulkarni, 2010). Determination of the phase purity, crystallinity, structure, particle size, elemental composition, microstructure, and functional group characterization of both the commercially prepared nanodiamonds (C_ND) and Ayu_ND samples was performed by XRD, FESEM with EDS spectroscopy, HRTEM, Raman spectroscopy, FTIR, and UV–VIS absorbance based on our previously described protocol (Srivastava et al., 2018). The details are in the methodology section of the supporting information.

### Animals, Cells and Generation of Solid Tumors

Female CD-1 [Crl:CD1(ICR)] mice, 6–8 weeks of age, were purchased from Hylasco Bio-Technology Pvt. Ltd, Hyderabad. Animals were housed in pathogen-free conditions of the institutional animal facility in accordance with the CPSEA guidelines. The culture of Dalton’s lymphoma (DL) cells and 2PK3 (murine lymphoma cell lines purchased from NCC, Pune) was performed as reported by us earlier. DL is a spontaneous murine lymphoma and was also maintained in the peritoneum with periodic transfer of the tumor cells to female CD1 mice. DL solid tumors were generated in female CD1 mice based on a previously described protocol (Sk et al., 2021). The details are in the methodology section of the supporting information.

### Isolation and Characterization of DCs and T Cells From the Spleen of CD1 Mice

Mouse splenic myeloid DCs (CD11c, MHC Class II, CD11b and CD8α) and T cells from normal, tumor-bearing or disease-free treated mice were isolated as described previously (Hira et al., 2014). Purified CD4^+^ or CD8^+^ T cells were isolated as described previously (Srivastava et al., 2017; Hira et al., 2020). Isolated DCs were further incubated with Ayu_ND (1 mg/ml) or LPS (5 mg/ml) for *in vitro* stimulation.

### Analysis of TNF-α Expression in DCs

Cell-free culture supernatant was generated from DCs stimulated with or without Ayu_ND and LPS for 16 h. The culture supernatant generated was kept at −80°C before the estimation of DC-derived secretory TNF-α by ELISA. DCs were analyzed by FACS analysis to measure membrane-bound TNF-α expression in DCs.

### Assessment of *In Vitro* DC-Mediated Cytotoxicity, Antitumor Activity and Apoptosis

Cell viability, antiproliferative effects, and cytotoxicity against tumor cells in the presence of Ayu_ND were studied by MTT and XTT assays, as previously described (Srivastava et al., 2018). DC-mediated cytotoxicity and antitumor activity were studied by MTT and cytotoxicity assays, as described previously (Hira et al., 2014). Details on the assays are presented in the supporting information. Evaluation of apoptotic cell death in DL by activated DCs or by nanodiamond formulations against DCs was assessed by binding FITC-conjugated Annexin-V as described earlier (Srivastava et al., 2017).

### *In Vivo* Therapeutic Study

*In vivo* therapy with Ayu_ND in DL solid tumor-bearing animals has been elaborately described and is based on a previously described protocol (Sk et al., 2021).

### Secondary Tumor Challenge

To determine the persistence of tumor-specific immunity in the mice treated with Ayu_ND, mice showing complete regression of DL solid tumors were given a second subcutaneous tumor challenge (5 × 10^6^ DL) in the left lower flank at day 90 after the first tumor inoculation (contralateral to the first injection site). These mice, as well as the fresh control group receiving the same number of tumor cells (5 × 10^6^), were monitored for tumor size and survival.

### Histopathological Analysis

Tumors, liver and spleen from the untreated and treated groups were removed at day 24. Tissue specimens were fixed in 10% neutral buffered formalin overnight, cut into 5 μm thick sections, and stained with hematoxylin/eosin or anti-CD8 antibody as described earlier (Sk et al., 2021).

### Antigen-Specific T-Cell Proliferation and Cytotoxicity Assay by CD8^+^ T Cells Derived from Treated Mice

T cells (CD4^+^) from healthy, tumor-bearing or vaccinated mice were used as responder cells against whole tumor lysate (10 μg/ml)-pulsed and mitomycin C (10 μg/ml)-treated stimulator DCs isolated from the treated mice. The MTT assay was used to determine cell proliferation as described previously (Hira et al., 2014). CTL assays were performed as reported previously (Hira et al., 2020). The details are in the methodology section of the supporting information.

### *Ex Vivo* Analysis of DCs and T Cells Following Therapy

DCs or T cells derived from normal, tumor-bearing (32 days post-tumor transplant) or vaccinated mice were stained for phenotypic analysis (i.e., surface expression of DC-specific markers, Treg status, CD8^+^ memory cell status, etc.) by flow cytometry as describe earlier (Hira et al., 2020). The details are in the methodology section of the supporting information.

### Immunofluorescent Staining and Cytokine ELISA

Immunofluorescent staining of CD3, CD8, and PD-1 was carried out on serial paraffin sections as described earlier (Hira et al., 2020). Serum was collected from each group, including naïve mice, and assayed for the presence of cytokines (TNF-α, IFN-γ and TGF-β) by sandwich enzyme-linked immunosorbent assay (ELISA) using a BioLegend ELISA Max^™^ assay kit according to the manufacturer’s protocol (Hira et al., 2014).

### Statistical Analysis

The mean ± standard deviation (SD) value was calculated for each experimental group (*n* = 3–5). Flow cytometry data were analyzed with FlowJo software (version 10.0.5; Treestar). Differences between or among the groups were analyzed by ANOVA followed by Holm-Sidak *post hoc* multiple comparison tests with the use of PRISM statistical analysis software (GraphPad). Survival plots (Kaplan–Meier) were generated with GraphPad Prism software, and statistical significance was analyzed by means of the Mantel–Cox log-rank test. A *p* value <0.05 was considered statistically significant.

## Results

### Analysis of Ayu_ND Modification and Toxicity

**FIGURE 1:**
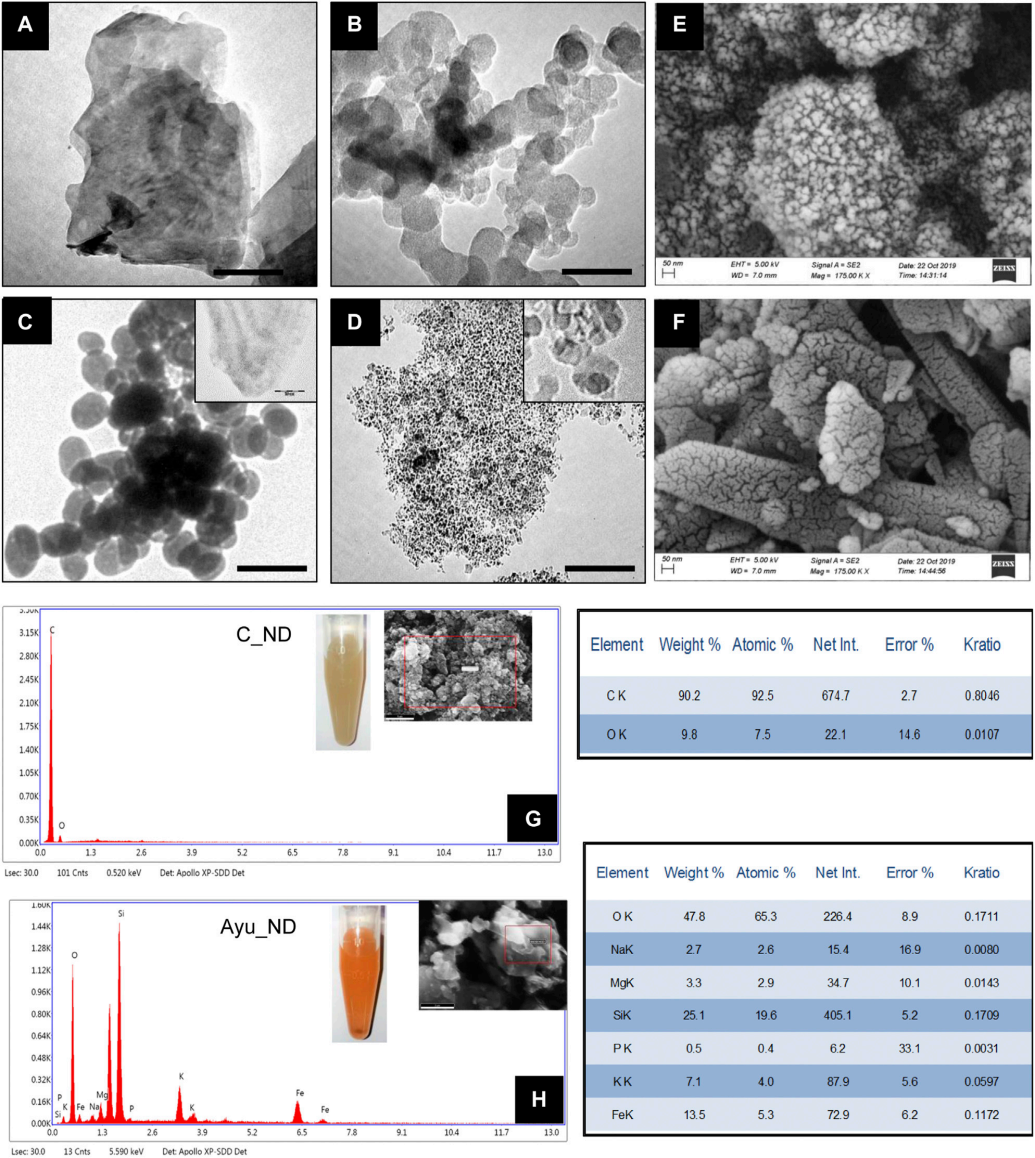
Characterization of as-prepared Ayu_ND. TEM images of the diamond powder **(A)**, diamond ash **(B)**, Heeraka Bhasma or Ayu_ND **(C)**, and commercial nanodiamond or C_ND **(D)**. The Scale bar is 50.0 nm. FESEM images of Ayu_ND **(E)** and C_ND **(F)**. Elemental analysis (EDAX), including the images (inset) of C_ND **(G)** and Ayu_ND **(H)** in aqueous solution. Representative images and plot of three independent experiments.

A physiochemical study of Ayu_ND reveals some new fascinating aspects of its properties that support its active biological functions. TEM studies revealed that Ayu_ND comprised large particles as well as nanosized diamond nanoparticles (Figures 1A–C). This is unique compared with commercially available nanodiamonds, which are powdery in nature (Figure 1D). The nanosized Ayu_ND nanoparticles were spherical and averaged ∼100 nm in size (Figure 1C). FESEM micrographs and EDAX analysis of Ayu_ND reveal that it belongs to the group of mineral powders that are granulated, unequal and unarranged in structure (Figures 1E,H). Elemental analysis of C_ND (Figures 1F,G) and traditionally prepared spherical diamond nanoparticles Ayu_ND by FESEM-EDAX shows that C_ND contains C and O, whereas Ayu_ND, in addition to C and O, is conjugated by Si, Na, Mg, P, K and Fe in different proportions. Similar observations were also found in the XRF analysis (Supplementary Figures S2A,B).

**FIGURE 2:**
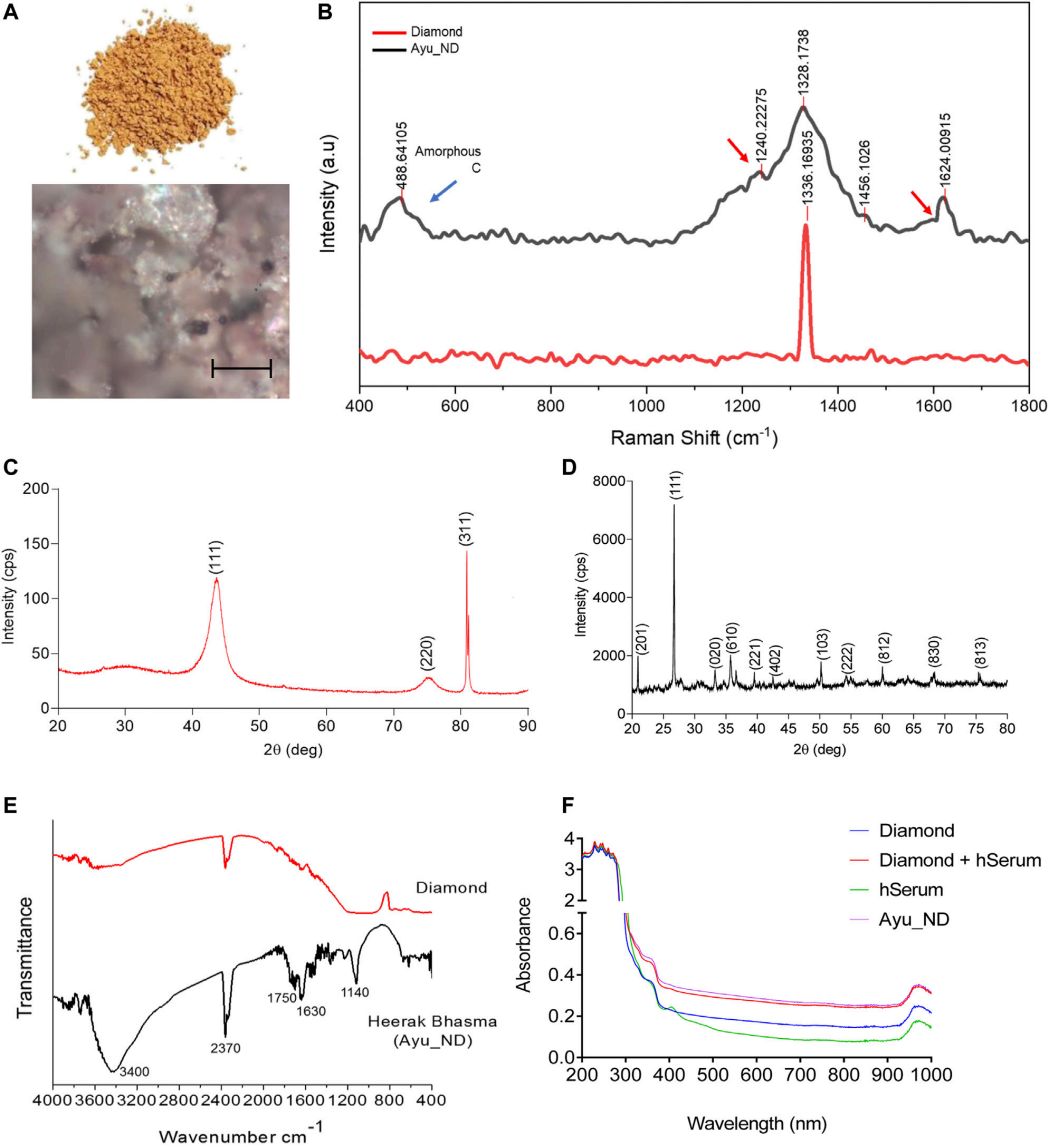
Spectroscopic analysis of Ayu_ND. Representative Optical images of three similar experiments are shown **(A)** and Raman spectra of diamond powder only (red) and after the formation of Ayu_ND (black) **(B)**. X-ray diffraction pattern of the diamond powder only **(C)** and formed Ayu_ND nanomaterial **(D)**. FTIR spectra of diamond powder only (red) and Ayu_ND (black) **(E)**. UV absorption spectra of free C_ND, human serum, human serum absorbed C_ND and Ayu_ND **(F)**. The experiment was repeated independently three times to confirm the results.

Raman spectroscopy is the most sensitive for highly symmetric covalent bonds with little or no natural dipole moment. Raman spectroscopy analysis of C_ND and Ayu_ND shows that the band of Ayu_ND matched that of pure diamond at 1333 cm^−1^ along with other peaks at 488.64 cm^−1^ and 1624 cm^−1^ (Figures 2A,B). The extra bands of Ayu_ND in the Raman spectrum manifested a slightly downshifted tetrahedral sp3 band due to the small crystal size of nanocrystalline Ayu_ND, which results in a finite-size effect in which the lattice is somewhat distorted. The additional band at 1624 cm^−1^ and the shoulders (red arrow) on the 1624 cm^−1^ and tetrahedral sp3 bands are also indicative of sp2 bonded carbon that represents surface defect modes, which would be insignificant in larger diamond crystals. Finally, the very broad band at approximately 488.64 cm^−1^ is indicative of some amorphous sp3 bonded carbon. XRD patterns of the C_ND powder mixture and Ayu_ND are shown in Figures 2C,D. In Figure 2C, the diffraction peaks of the diamond powder showed three peaks (111, 2θ = 41.74), (220, 2θ = 75.8) and (311, 2θ = 82.1), demonstrating the cubic crystal of diamond. The grain size of the diamond powder was determined to be 7.58 nm, which was calculated by Scherrer’s equation.

The stronger background in the lower angle region for the whole spectrum suggests that there is certain amorphous material in the diamond powder. In Figure 2D, diffraction peaks corresponding to diamond are observed for the Ayu_ND sample along with other peaks, and the results demonstrate that Ayu_ND presents an orthorhombic crystal structure. The average particle size of Ayu_ND was calculated to be 25.9 by Scherrer’s formula. Thus, it is clear that the crystal structure of Ayu_ND is changed due to ayurvedic processing. The crystal phase analysis showed that Ayu_ND contains carbon that occurs predominantly in the diamond phase. The other main elemental components are silicon, iron, calcium, sodium and magnesium. The phase pattern identified from the XRD result agreed with the EDS result, as shown in Figure 1H.

Several studies have shown that high-pressure-high temperature nanodiamonds (HPHT-NDs) can bind noncovalently but strongly with proteins through a combination of electrostatic forces, hydrogen bonding, and hydrophobic interactions. Ayu_ND was subjected to PAGE analysis, resulting in a distinct protein band of ∼91 KD (Supplementary Figure S2C). The presence of protein in Ayu_ND suggests that during synthesis, proteins from human serum albumin bind noncovalently with the nanodiamonds. To confirm this, UV–VIS analysis of the nanodiamond powder, Ayu_ND, human serum and human serum mixed with nanodiamond powder indicated protein attachment on Ayu_ND (Figure 2F). To evaluate the possible use of Ayu_ND as a therapeutic agent *in vivo*, we evaluated the biological responses of Ayu_ND at higher dosages. As shown in Supplementary Figure S3A, the Ayu_NDs, even at high concentrations (100 μg/kg body weight), did not induce any systemic inflammatory responses; as a result, serum interleukin-6 (IL-6) concentrations remained unaltered. Furthermore, Ayu_ND treatment did not result in any increase in serum alanine transferase (ALT), suggesting that Ayu_ND treatment does not adversely affect liver functions (Supplementary Figure S3B). Histological analysis revealed no significant changes in multiple tissues that were analyzed (Supplementary Figures S3C–F). A biocompatibility study of C_ND or Ayu_ND suggests that Ayu_ND is relatively tolerant to lymphocytes and monocytes derived from splenocytes and normal NIH/3T3 cells (Supplementary Figures S4A–C). In addition, Ayu_ND induced low to negligible hemolysis in a concentration-dependent manner, suggesting tolerance to RBCs (Supplementary Figure S4D). These initial biocompatibility studies indicate that NDs may be applicable as therapeutic agents *in vivo*.

### Ayu_ND Activates Mouse Dendritic Cells and Augments DC-Mediated Antilymphoma Activity *In Vitro*

In the Ayurvedic text, *Heerak Bhasma* (also called *Vajra Bhasma, Hira Bhasma and Diamond Bhasma*) is mainly used for the treatment of internal abscesses, tumors, cancer, angina pectoris and tuberculosis (Pal et al., 2014; Sharma, 2014). *Heerak Bhasma* may act as an immunomodulatory agent and strengthen the mind and body (Sharma, 2014). To assess the immunomodulatory activity of Ayu_ND, we chose dendritic cells (DCs) since these unique cells act as professional antigen-presenting cells and killer cells based on their activation stimuli (Hira et al., 2020).

**FIGURE 3:**
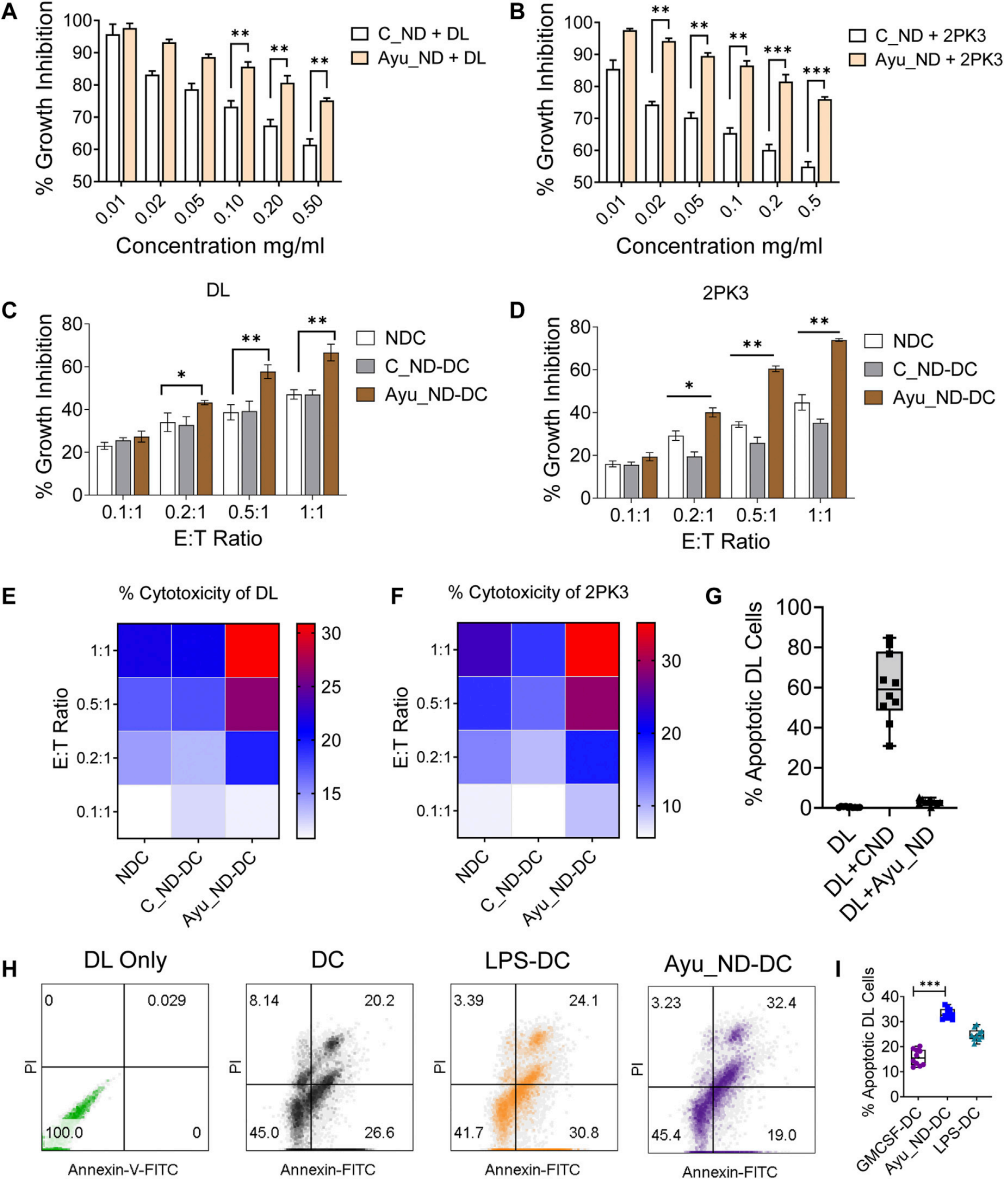
Ayu_ND augmented DC-mediated antitumor effects against murine lymphoma. Ayu_ND alone is tolerant against DL and 2PK3 murine lymphoma cells. DL **(A)** and 2PK3 **(B)** cells were treated with different concentrations of C_ND or Ayu_ND for 48 h, and growth inhibition was assessed by using the MTT assay. The results are presented as the mean ± SD, *n* = 4. Naïve, C_ND or Ayu_ND in the presence of naïve or activated splenic DCs were cocultured with lymphoma cells (DL) **(C)** and 2PK3 **(D)** at different E:T ratios for 48 h followed by MTT assay. An 18 h cytotoxicity assay was performed using naïve and activated DCs against DL **(E)** and 2PK3 **(F)** by LDH release assay. The heatmap color scale indicates the relative enhancement of cytotoxicity (*n* = 3). Apoptosis in DL cells following culture with Ayu_ND and CNDs **(G)** or Ayu_ND-activated DCs **(H)** was analyzed by FACS following staining with Annexin FITC and PI. % Apoptotic DL cells were documented by flow cytometer analysis **(I)**. Significance was determined by an unpaired Wilcoxon test with Benjamini and Hochberg correction: *p < 0.05, **p < 0.01, ***p < 0.001, and ****p < 0.0001, *n* = 6.

Our results indicate that C_ND treatment significantly affects the viability of tumor cell growth *in vitro*, while Ayu_ND is unable to exert a similar impact (Figures 3A,B). On the other hand, DC-mediated *in vitro* growth inhibition of DL and 2PK3 cells was significantly enhanced when DCs were activated with Ayu_ND. Compared to naïve or C_ND DCs, DCs stimulated with Ayu_ND acquired significant growth inhibition and cytotoxicity potential against DL and 2PK3 cells in a dose-responsive manner (Figures 3C–F). Ayu_ND had a more effective stimulatory response than the traditional treatment with GM-CSF (Supplementary Figure S5). LPS-treated DCs were used as a positive control for DC activation. Furthermore, Ayu_ND-activated DCs induced greater apoptosis of DL cells, suggesting that Ayu_ND-activated DCs can be a better immune adjuvant to mount an effective immune response against tumor cells (Figures 3G–I).

**FIGURE 4:**
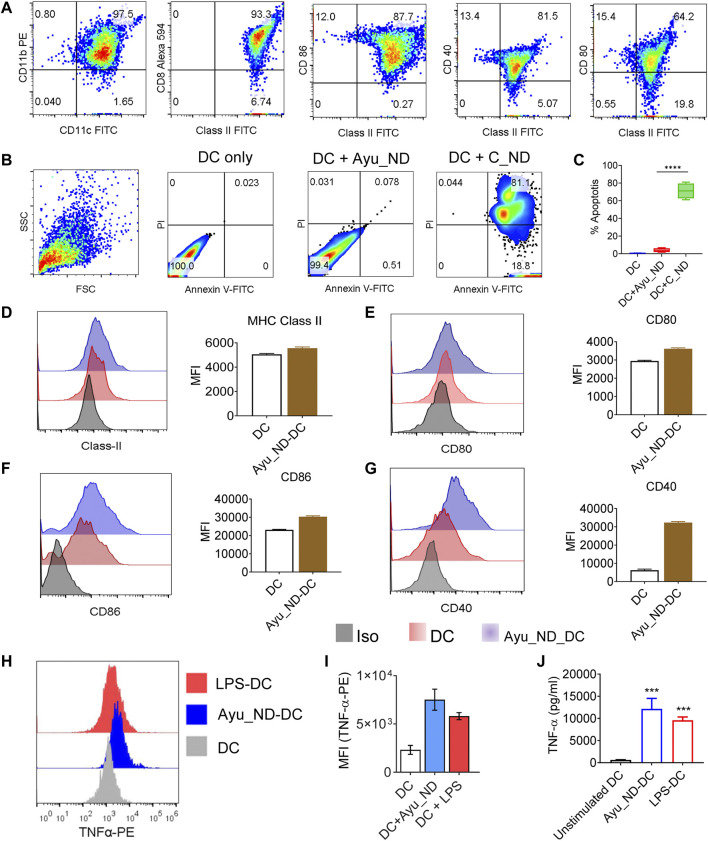
Ayu_ND induces dendritic cell activation and enhances DC-derived TNF-α expression. CD11c+/CD11b+ DCs were assessed by flow cytometry for the expression of MHC class II, CD80, CD86 and CD40 **(A)**. DCs were treated with either medium alone or with 200 μg/ml Ayu_ND or C_ND **(B)** for 120 h, and the percent apoptosis was determined by FACS analysis indicated in the blue boxes of the double-positive quadrate. % Apoptotic DCs were documented by flow cytometric analysis **(C)**. The expression of MHC class II, CD80, CD86 and CD40 was assessed by flow cytometry in 10 μg/ml Ayu_ND-treated CD11c+/CD11b+ DCs **(D**–**G)**. The mean fluorescence intensity (MFI) of class II and costimulatory molecules in dendritic cells following treatment with Ayu_ND is presented. DCs were either treated with medium only or treated with 10 μg/ml Ayu_ND or LPS (5 μg/ml) to determine the expression of TNF-α by flow cytometry **(H)**. MFI of TNF-α expression in the three-groups **(I)**. Estimation of TNF-α by ELISA in the culture supernatants of DCs following the indicated treatment **(J)**. The experiment was repeated independently four times (*n* = 4) to confirm the results. Represtative dot plot and histogram plots are presented here. In bar diagarms, data are presented as the mean ± SD, *n* = 6. *p < 0.05, **p < 0.01, ***p < 0.001, and ****p < 0.0001, *n* = 6. *In vivo* inhibition or regression of long-term tumor growth by Ayu_ND generates strong CD8^+^ T cell immunity.

Activation of CD11c^+^/Class II^+^ DCs was derived from the spleen of healthy animals (Figure 4A), and treatment with C_ND substantially reduced the viability of DCs and induced apoptosis (Figures 4B,C). Treatment with Ayu_ND and DCs showed remarkable tolerance in addition to improved functional aspects, which were connected to improved immune responses. CD11c^+^/Class II^+^ DCs showed enhanced expression of class II and costimulatory molecules such as CD40/CD80/CD86, indicating upregulation of the immune response in DCs when activated with Ayu_ND (Figures 4D–G). Intracellular TNF-α expression in DCs was also assessed to show the cytokines that regulate the effector functions of DCs for tumor immunity. TNF-α expression in DCs was increased substantially upon activation with Ayu_ND (Figures 4H–I). LPS-treated DCs were used as a positive control for DC activation. TNF-α was also substantially present in the culture supernatant compared with the untreated culture supernatant (Figure 4J). The upregulation of the above molecules explained the improved and prodigious DC-mediated immune responses in the presence of Ayu_ND against lymphoma cells. To further define the role of Ayu_ND activated DC derived TNF-α mediated antitumor effects against lymphoma cells *in vitro* we performed antibody neutralization experiments (Supplementary Figure S6). The approach identified that the Ayu_ND-activated DC-mediated antitumor effect is mediated by DC-derived TNF-α.

**FIGURE 5:**
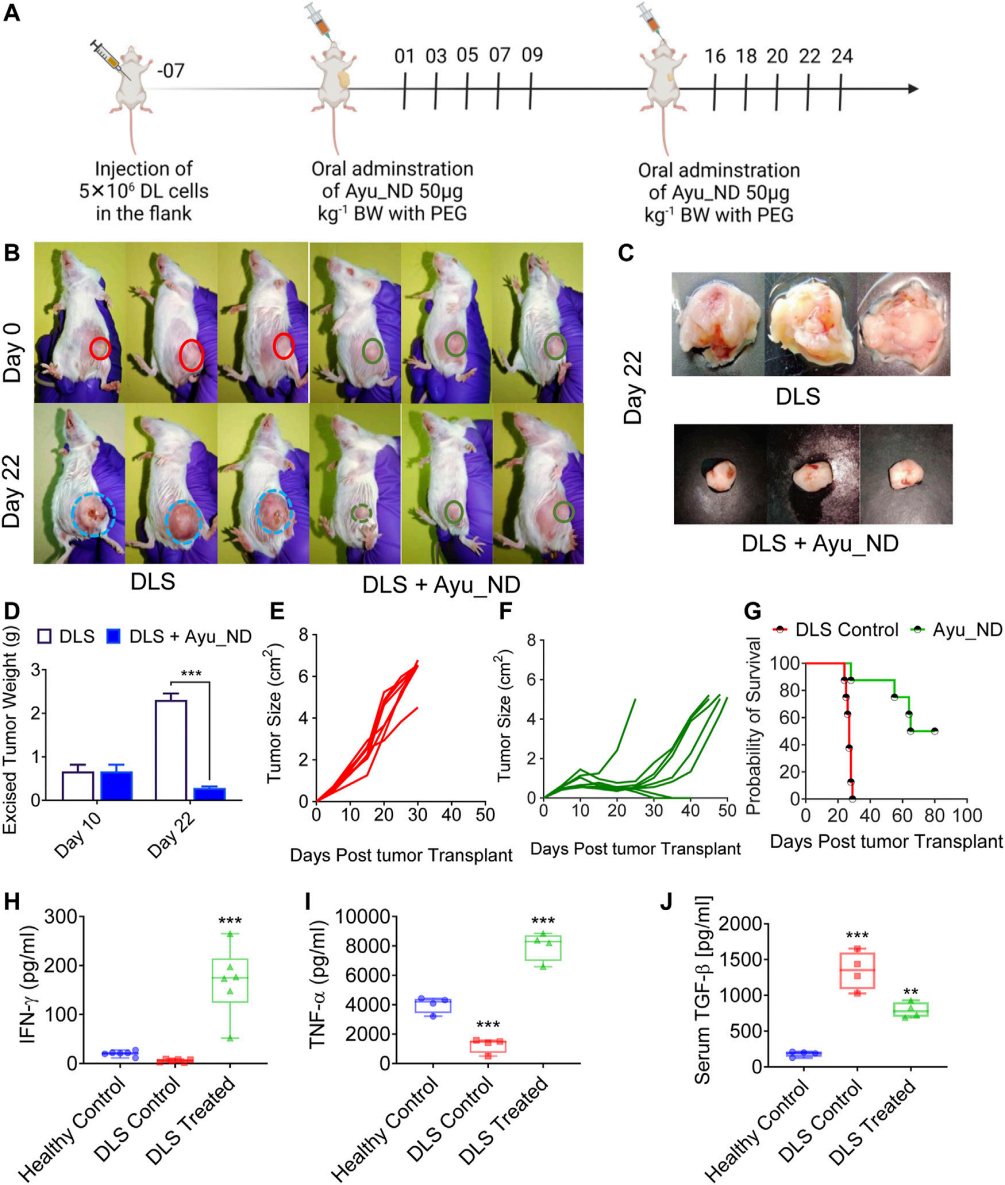
Ayu_ND treatment suppresses the growth of DL solid tumors and enhances long-term survival. Scheme of the therapeutic protocol illustrating the time points of Ayu_ND administration in DL solid tumor-bearing CD1 mice. Ayu_ND (50 μg/kg) was orally administered on days 1, 3, 5, 8, 7, and 9 and on days 16, 18, 20, 22 and 24. Tumor volume was measured on days 2, 4, 8, 12, 16, 20, and 24 and continued until day 50 in the treated group **(A)**. On day 22, three mice from each group were sacrificed between 9:00 a.m. and 11:00 a.m. The tumors were obtained. Images of solid DL tumor growth in CD1 mice at day 0 (before treatment) and after 3 weeks (day 22) following treatment with Ayu_ND **(B)**. Tumor weight comparison between untreated and treated groups performed at day 22 **(C**,**D)**. Tumor size in the untreated and Ayu_ND-treated groups showed significantly reduced tumor volume in the treated group compared with the untreated control at day 22 **(E**,**F)**. Kaplan–Meier survival analysis of the tumor-bearing mice following the abovementioned treatment with Ayu_ND **(G)**. Serum levels of IFN-γ **(H)**, TNF-α **(I)** and TGF-β **(J)** in Ayu_ND-treated animals compared with untreated animals at day 22. Statistical significance was analyzed by two-tailed Student’s t test **(C**,**D)** or by the log-rank (Mantel–Cox) test **(E)**. NS no significance; *p < 0.05, **p < 0.01, ***p < 0.001, and ****p < 0.0001.

The solid tumor model system is the most reliable representative to study the major histological parameters in cancer. We used a highly metastatic murine lymphoma called Dalton’s lymphoma (DL). We were successful in making the DL tumor grow as a solid tumor in the right flank of the female CD1 mouse (∼4–6 weeks old) (Srivastava et al., 2018). Tumor formation under the skin was palpable after 8–10 days, and the tumors grew and formed large tumor masses (Figure 5). A therapy schedule was formulated involving Ayu_ND (50 μg/kg body weight) in tumor-bearing mice (Figure 5A). Individual images show DL solid tumors in CD1 mice before (day 0) and 3 weeks (day 22) after treatment with Ayu_ND (Figure 5B). The tumor weights of the control and Ayu_ND groups were 2.30 ± 0.14 and 0.283 ± 0.035 g, respectively, and a significant difference was observed between the two groups (*p* < 0.01) (Figures 5C,D). The tumor volume or size of the Ayu_ND group was significantly reduced compared with that of the control group (*p* < 0.05). The results of the scatter graph of solid tumor weight are presented in Figures 5E,F. Furthermore, the survival time in the Ayu_ND-treated group was considerably increased compared with that in the healthy control group (*p* < 0.05) (Figure 5G). We also studied the anti- and proinflammatory cytokine profiles of the treated group to correlate the therapeutic outcome with cytokine involvement. Our data suggest that serum IFN-γ and TNF-α levels were high in the Ayu_ND-treated group relative to the downregulation of these molecules in tumor-bearing animals (Figures 5H–J). These results suggest that IFN-γ acts as a healer agent for ND therapy. In contrast, TGF-β levels increased in tumor-bearing animals and decreased to basal levels in the treatment group (Figure 5J). In line with the observation made by others (Bitzer et al., 2000; Yamane et al., 2003), our data suggest that IFN-γ and TNF-α impaired the response of the tumor cells to TGF-β.

**FIGURE 6:**
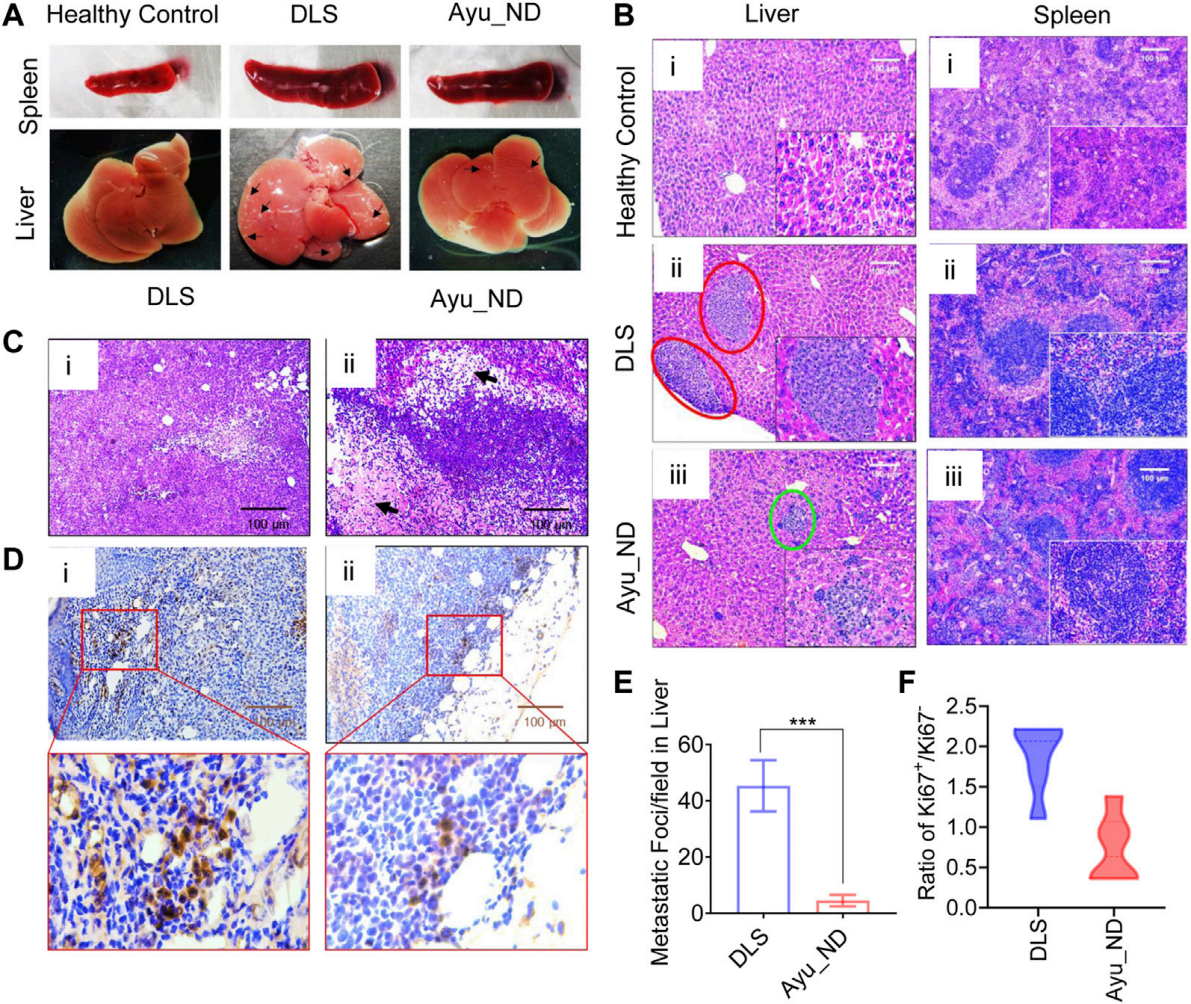
Histopathological features of Ayu_ND-treated animals show disease protective potential. Macroscopic liver metastatic nodule distribution in the dissected organs (indicated with black arrow) **(A)**. Representative H&E-stained livers **(i**–**iii)** and spleens **(i**–**iii)** collected from healthy control, untreated and treatment groups at day 22 and labeled metastatic foci areas indicated with red circles for DLS and green circles with Ayu_ND treatment **(B)**. H&E-stained sections of solid tumors **(C)**. Staining of solid tumor sections with anti-mouse Ki67 antibodies for detecting dividing cells for comparisons between the untreated and Ayu_ND-treated groups **(D)**. Quantitative estimation of metastatic foci in the livers of untreated and treated mice **(E)**. Ratios of Ki-67+/Ki-67− tumor cells in the solid tumor mass between the treated and untreated groups **(F)**. Data represent the mean ± SE of *n* = 3 or 4 animals per group. NS no significance; *p < 0.05, **p < 0.01, ***p < 0.001, and ****p < 0.0001.

Physical examination of normal, untreated tumor-bearing and treated mice showed that DL cells infiltrated and mostly replaced the subcutaneous tissue (Figure 6A). Representative images are shown in Figure 6A. Histopathological analysis of the liver and spleen showed extensive metastasis with the tumor cells in the untreated animals, while the treated group appeared to have cleared large portions of such infiltration (Figure 6B). Quantitative estimation of tumor foci in the liver demonstrated a significant difference in the number of metastatic foci between the untreated and treatment groups (Figure 6E). Examination of the tumor showed numerous newly formed blood capillaries (neovascularization) in the surrounding tissues with mild or no inflammatory responses (Figure 5C). Such tumor histology showed tissue architectural disarray, as well as a marked degree of cellular anaplasia, pleomorphism, and anisocytosis, with nuclear vascularity, atypicality, hyperchromasia, and mitoses. Minimum necrotic areas with pyknosis and karyolysis appeared in the central regions of the tumors in the form of round-cell infiltrations and hemorrhages (Figure 6Ci [if subparts are of C]).

In contrast, mice treated with Ayu_ND showed minimal tumor cells, extensive necrosis, and apoptosis at the margin of the tumor with damaged blood vessels with hemorrhage. High numbers of round cells of large lymphocytes and macrophages invaded the necrotic zones (Figure 6Cii [if subparts are of C]). To evaluate the antiproliferative effect of Ayu_ND, Ki-67 (a cell proliferation marker) immunohistochemistry staining was performed to quantify cell proliferation in the tumor sections from all the groups (Figure 6D). The ratio of Ki67^+^/Ki67^-^ was calculated by counting the cells from 10 high-power fields randomly selected from each treatment group. The ratio of Ki67^+^/Ki67^-^ of the Ayu_ND group was decreased significantly in treated animals compared with untreated littermates (Figure 6).

### Decreased Treg/Th Balance and Systemic Immune Response by Ayu_ND Treatment

**FIGURE 7:**
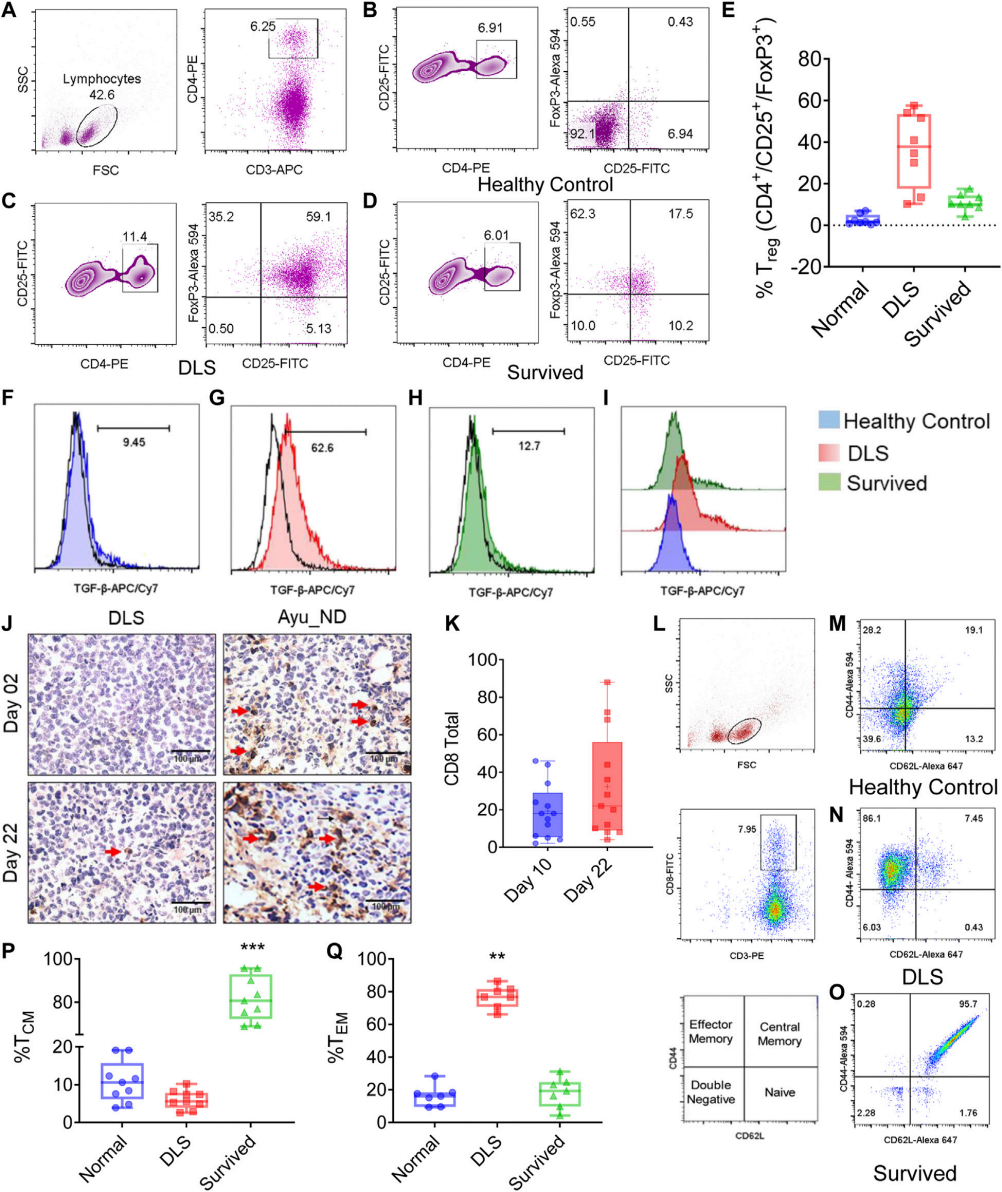
Ayu_ND abrogates regulatory T cells and enhances vaccine-mediated CD8^+^ T-cell immunity. Splenocytes from healthy control, DLS tumor-bearing and tumor-free mice were stained for CD3^+^CD4^+^CD25^+^Foxp3^+^ Treg cells. The cells were gated on CD4^+^CD25^+^ T cells **(A)**. The percentage of CD25^+^Foxp3^+^ cells is presented in the upper right quadrant of each plot for the indicated groups **(B**–**D)**. The percentages of CD25high and FoxP3^+^ cells in CD4^+^ T cells are shown in representative box plots for healthy control, untreated and Ayu_ND-treated mice **(E)**. Reduction in TGF-β secretion *in vivo* in CD4^+^CD25^+^Foxp3^+^ Treg cells following Ayu_ND treatment compared to the untreated control **(F**–**I)**. Sections of the solid tumor were stained with anti-mouse CD8 antibodies **(J)**. Quantitative estimation of the tumor-infiltrating CD8^+^ T cells in five randomly chosen areas per slide in three slides per tumor and presented as the mean ± SD **(K)**. Gating strategy of CD3^+^/CD8^+^ T cells from splenocytes and representation of various T-cell memory subsets: naïve (TN) (CD44^−^^CD62 L+^), central memory (T_CM_) (CD44^+ CD62 L+^), effector memory (T_EM_) (CD44^+^^CD62 L+^), and double negative (TDN) (CD44^−^^CD62 L−^) phenotypes **(L**–**O)**. Frequencies of central and effector memory T cells in untreated and Ayu_ND-treated mice **(P**,**Q)**. Data represent the mean ± SE of *n* = 3 or four animals per group. NS no significance; *p < 0.05, **p < 0.01, ***p < 0.001, and ****p < 0.0001. Immunity induced by Ayu_ND treatment is long-lived.

The reduction in TGF-β in the serum of the Ayu_ND-treated group prompted us to investigate the role of regulatory T cells (Tregs) in treated animals. We examined the feasible connection between the clearance of tumor cells and the alteration of FOXP3^+^ Tregs in the T_h_ cell repertoire. Quantitative estimation indicated that CD4^+^ T cells were overwhelmed in DL mice and noticeably diminished in the Ayu_ND-treated group (Figures 7A–E). We also examined the CD4^+^CD25^+^FOXP3^+^ population in the T cells in untreated mice and mice treated with Ayu_ND. CD4^+^CD25^+^ cells were reduced from 11.4% in untreated mice to 6.01% in animals treated with Ayu_ND. When we examined CD25^+^FOXP3^+^ cells, we observed a massive surge in this population (59%) in untreated DL mice, which decreased to less than 18% in mice that received Ayu_ND (Figure 7E). Ayu_ND treatment limits FOXP3-positive regulatory T cells and downregulates TGF-β synthesis compared with untreated mice (Figures 7F–I).

### Ayu_ND Promotes the Differentiation of CD8^+^ Effector Memory T Cells

We examined the infiltration of CD8^+^ T cells in untreated and treated animals (Figure 7J). The data suggest a dramatic reduction in tumor-infiltrating CD8^+^ T cells in the tumor, which was significantly increased in Ayu_ND-treated mice. The tumor-infiltrated CD8^+^ T cells were calculated by counting the cells from 10 high-power field areas randomly chosen from each treatment group. Representative images are shown in Figure 7K. To show the memory phenotype of the CD8^+^ T cells in the spleen of the treated animals, we performed FACS analysis of splenocytes. CD8^+^ T cells gated on CD3^+^ T (Figure 7L) cells were double stained with CD44 and CD62 L. CD44, whose isoforms are widely and asymmetrically expressed in breast carcinoma and correlated with tumor subtypes and cancer stem cell markers, has a similar role in other types of cancer (Ponta et al., 2003; Zöller, 2011; Chen et al., 2018). Our results suggest that after treatment with Ayu_ND, T cells expressed CD62 L, which was nearly absent in untreated DL mice (Figures 7M–O). We also compared the percentages of effector memory T cells (T_EM_ cells) and central memory T cells (T_CM_ cells) based on the differential expression of selectin. Both CD4^+^ and CD8^+^ T cells have two main subclasses of memory cells: central memory (T_CM_) and effector memory (T_EM_) T cells (Sallusto et al., 2004). T_EM_ cells are phenotypically different from T_CM_ cells, and they generally express low levels of CD62 L and CD127 and high levels of KLRG-1 and are deficient in CCR7. In contrast to T_CM_ cells, T_EM_ cells manifest quick and extensive effector functions involving the production of granzyme B and IFN-γ, although they have limited proliferative response potential (Jameson and Masopust, 2009). Our results suggest that in DL tumor-bearing animals, splenic T cells consist of the T_EM_ type with low expression of CD62 L, which was reduced significantly by Ayu_ND (Figure 7P). In contrast, Ayu_ND-treated mice had T_CM_ cells, and DL tumor-bearing animals had very low or no CD62 L expression (Figure 7Q).

**FIGURE 8:**
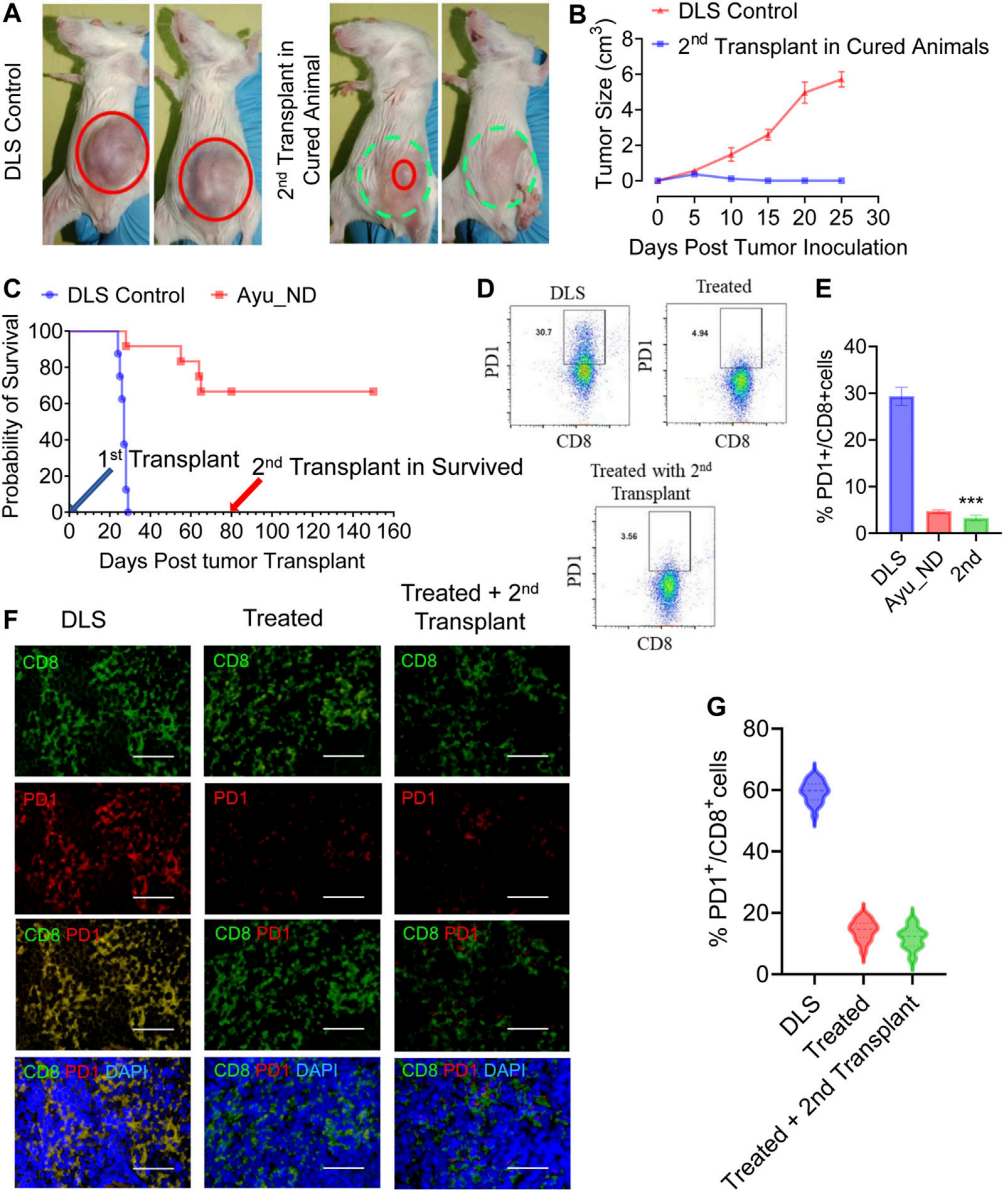
Immunity induced by Ayu_ND treatment is long-lived. The growth of subcutaneous tumors in naïve control mice and mice survived and rechallenged >2 months after primary tumor rejection **(A)**. Complete nonappearance of a tumor is displayed **(B)**, and enhanced survival was observed with further treatment **(C)** in the case of rechalange experiments. The number of PD1-expressing CD3^+^CD8^+^ T cells was higher in the untreated mice than in the other groups of tumor-draining lymphocytes **(D**,**E)**. Paraffin sections derived from untreated, treated with Ayu_ND or rechalanged mice after curing with Ayu_ND treatment were stained with anti-mouse PD-1 (red) and counterstained with anti-mouse CD8 (green) and DAPI for nuclei (blue). Colocalization of these two markers was detected by merging the monostaining pictures (*n* = 5). Scale bar, 50 mm **(F)**. Quantitative estimation of the percentages of PD-1^+^ and CD8^+^ T cells is presented in the bar graph (*n* = 5) **(G)**. Data represent the mean ± SE of *n* = 3–5 animals per group. NS, no significance; *p < 0.05, **p < 0.01, ***p < 0.001, and ****p < 0.0001.

To assess the memory response, we observed that mice (Figure 8) treated with Ayu_ND were rechallenged with DL cells, and naïve mice were challenged in parallel. No tumor growth was observed in the rechallenged group, indicating a memory response upon tumor antigen recognition. In contrast, naïve mice developed large, progressively growing tumors. The complete absence of a tumor and increased survival (Figures 8A–C) without any further treatment in the rechallenge group indicates that the immunity induced by Ayu_ND treatment is long-lived and tumor specific. The success of Ayu_ND treatment restored the immune responses in treated animals. In addition to changing the cellular topography in the tumor-bearing mice, CD4^+^ T cells in the spleen demonstrated antigen-specific proliferation derived from the animals that received Ayu_ND treatment (Supplementary Figures S7A–C). We also examined the potential of CD8^+^ T cells to directly kill target cells. CD8^+^ T cells from tumor-bearing mice are greatly impaired in cytotoxicity against DL target cells. CD8^+^ T cells from the animals treated with Ayu_ND restored the potential and augmented cytotoxicity against DL tumor cells (Supplementary Figures S7D,E).

Likewise, T cells and dendritic cells (DCs) also had considerable improvements in their functional aspects, which indicates revised immune responses in the treated group. CD11c^+^/Class II^+^ DCs, derived from the animals treated with Ayu_ND*,* increased in number significantly compared with the untreated littermate (Supplementary Figures S8A–H). The expression of class II and costimulatory molecules such as CD40/CD80/CD86 showed significant upregulation in animals that received treatment compared with untreated mice. The upregulation of the above molecules explained the improved and prodigious immune responses in tumor-bearing animals that received Ayu_ND therapy. Enhanced immune function of DCs was also evident in increased cytotoxic potential against DL tumor cells. Effector DCs (CD8^+^ killer DCs) from the spleen of the treated animals demonstrated significantly higher cytotoxicity against the target cells than DCs derived from untreated mice (Supplementary Figures S8I–K).

## Discussion

The Indian traditional medicine “Ayurveda” is the oldest indigenous medicine system of herbomineral drugs and is known to have antiquity effects in preventing or suppressing various tumors by boosting the immune system (Sharma, 2014). Currently, scientists are more than ever keen to find complementary and alternative medicines for the management of cancer. Ayurvedic formulations are multicomponent mixtures containing products derived from plants and animals, minerals and metals (Mukherjee and Wahile, 2006). Since ancient times, *Bhasmas* have been exclusive ayurvedic metallic formulations that are used for medicinal purposes (Umrani and Paknikar, 2011). *Bhasmas* are nontoxic, easily absorbable, adaptable and digestible in the body (Verma and Prasad, 1995; Singh et al., 2010; Umrani et al., 2013) if they are prepared properly. These *Bhasmas* are generally prescribed several other medicines prescribed in Ayurveda (Pal et al., 2014; Chaudhari et al., 2016). Ancient nanomedicine in the form of ayurvedic *Bhasmas* provides an opportunity for the safer use of living beings (Sarkar and Chaudhary, 2010; Chaudhary, 2011; Paul and Sharma, 2011; Jagtap et al., 2013; Pal et al., 2014; Kale and Rajurkar, 2018; Kantak et al., 2019). *Bhasmas* have some advantages over plant-based drugs, such as stability over longer periods, lower dosages, easy storability and easy availability (Kumar et al., 2006; Singh et al., 2010; Wijenayake et al., 2014).

In this work, we aimed to target experimental lymphoma using Ayurvedic nanodiamonds, which act as immunostimulators. A large number of studies have shown that nanodiamonds have been used for drug, protein, and gene delivery vehicles (Lam and Ho, 2009). Applications of these nanodiamonds were facilitated due to their unique properties, such as easy conjugation with bioactive ligands and high loading capacities per weight, as well as functional mechanisms for targeted release. Deagglomerated NDs play a significant role in the therapeutic release of biomolecules such as insulin and transforming growth factor beta (TGF-β) (Shimkunas et al., 2009; Smith et al., 2011). Additionally, this nanodiamond-delivered insulin is effective in wound healing and vascularization in patients with severe burns and other conditions and targets bacterial infections accompanying serious wounds.

In the present study, we designed a novel strategy for a fundamental understanding of the tumoricidal properties of nanodiamonds in close association with physiological cooperation with dendritic cells against malignant and metastatic lymphoma. We have elucidated some of the intriguing phenomena of nanodiamonds and detailed the nature and consequence of the protection provided by Ayu_ND. Our results demonstrated that ayurvedic nanodiamonds with the assistance of DCs increased tumoricidal properties against metastatic lymphoma generated as solid tumors in CD1 mice. Treatment with nanodiamond remarkably extends the life span of animals with active disease. This positive response was likely due to the positive tilting toward inflammatory cytokines such as TNF-α and IFN-γ, suggesting the generation of a solid T cell response during therapy. In addition, TNF-α is now also recognized for its capacity to inhibit the growth of tumor cells *via* activation of programmed cell death using the extrinsic pathway (Webster and Vucic, 2020).

Reports on the crosstalk between TGF-β signaling and TNF-α, showed that TNF-α impaired the response of the cells to TGF-β by regulating the turnover of TGF-β RII and activation of SMAD7 (Verrecchia et al., 2000; Yamane et al., 2003). Subsequent downregulation of serum TGF-β levels further suggests that treatment with nanodiamonds has a significant impact on the disease, exacerbating regulatory T cells. Significant elevation of regulatory CD4^+^CD25^+^ T cells marked the events in untreated DL mice that were ablated following therapy with nanodiamond. In addition to severely restricting the opulence of Treg cells and reducing TGF-β synthesis, nanodiamond treatment allowed the growth and differentiation of antigen-driven CD8^+^ T cells, as judged by the increase in Ki-67 staining in the spleen. This tumor protective immune response likely has a major impact on restricting tumor growth locally and preventing the dissemination of metastasis. This development occurs with the development of central memory T cells (T_EM_) with subsequent downregulation of central memory T cells (T_CM_). In nanodiamond-treated animals, the overwhelming presence of CD44^+^CD62 L^+^ memory T cells makes a significant difference in pathology and limits tumor growth, while untreated animals have loads of CD44^+^ T cells in lymphoid organs such as the spleen.

## Conclusion

This work demonstrates for the first time the immunostimulatory and tumoricidal properties of nanodiamonds-based ayurvedic herbomineral preparations against highly metastatic and aggressive murine lymphoma. The unique capacity of the Ayu_ND to activate dendritic cells suggests its potential to initiate strong, long-lasting adaptive killer cell immunity with a distinct memory phenotype. Strong interactions of monocrystalline nanodiamonds based ayurvedic formulations with the immune system usher a new understanding in therapeutics. This observation is unique and could provide a novel approach to address menacing problems, such as lymphoma, with serious impact. It is interesting to see that age-old therapeutic practice such as Ayurvedic nanodiamond initiates memory T cells response with effector phenotype suggesting, a key role and accommodate body’s effector functions to fight against diseases like lymphoma.

Ayurvedic nanodiamonds truly initiate an overwhelming tumoricidal immune response against highly metastatic lymphoma with poor prognosis, causing rapid death. However, reverse pharmacological analysis of these preclinical experimental findings may have a number of limitations since each patient is different, as is their disease course. Thus, the results provide a basis for further evaluation of the localization, entry of Heerak Bhasma (Ayu_ND) and impact on immunological parameters of human beings.

## Data Availability

The original contributions presented in the study are included in the article/Supplementary Material, further inquiries can be directed to the corresponding authors.

## Abbreviations

Ayu_ND, Heerak Bhasma; CD, cluster of differentiation; DC, dendritic cell; DL, Dalton’s lymphoma; DLS, Dynamic light scattering; DMSO, dimethyl sulfoxide; EDX, Energy Dispersive X-ray Analysis; ELISA, enzyme-linked immunoassay; FACS, Fluorescence-activated cell sorting; FBS, fetal bovine serum; FTIR, Fourier Transform Infrared Spectroscopy; IFN-γ, Interferon-TGF-2-β-transforming factor; IL, interleukin; LPS, lipopolysaccharide; MTT, (3-(4,5-Dimethylthiazol-2-yl); ND, Nanodiamond; NP, nanoplatinum; PBS, phosphate buffered saline; PI, propidium iodide; SEM, scanning electron microscopy; TEM, Transmission electron microscopy; TNFα, tumor necrosis alpha; XRD, X-ray diffraction; XTT, (2,3-Bis-(2-Methoxy-4-Nitro-5-Sulfophenyl).
