# Supplementary material for: Nanoscale Diamond-Based Formulation as an Immunomodulator and Potential Therapeutic for Lymphoma
Source: Front Pharmacol. 2022 Apr 4;13:852065. doi: 10.3389/fphar.2022.852065 (PMC9014173; doi:10.3389/fphar.2022.852065)
Supplement: Supplementary file 1 [file DataSheet1.PDF]

Nanoscale diamond-based formulation as an immunomodulator and potential therapeutic for lymphoma

Ankush Paladhi[1], Abhinandan Rej[1], Debanjan Sarkar[2], Ranjeet Singh[3], Sankar Bhattacharyya[2], Prasanta Kumar Sarkar[4], Pulak Kanti Kar[5], Partha Pratim Manna*[3], and Sumit Kumar Hira*[1]

[1]Cellular Immunology Laboratory, Department of Zoology, The University of Burdwan, Burdwan, India

[2]Immunobiology Lab, Department of Zoology, Sidho Kanho Birsha University, Sainik School, Ranchi Road, Purulia, West Bengal, India

[3]Immunobiology Laboratory, Department of Zoology, Institute of Science, Banaras Hindu University, Varanasi, India

[4]Department of Rasashastra, J. B. Roy State Ayurvedic Medical College and Hospital, 170-172, Raja Dinendra Street, Kolkata, West Bengal

[5]Department of Panchakarma, J. B. Roy State Ayurvedic Medical College and Hospital, 170-172, Raja Dinendra Street, Kolkata, West Bengal

* **Correspondence**: E-mail: pp_manna@yahoo.com (PPM) senior author and sumit.hira2008@gmail.com (SKH) Lead contact

Keywords: *Heerak Bhasma, Ayurveda, Nanodiamond, Lymphoma, Dendritic cell, T-cell*

Supplementary Table S1: List of Antibodies and Reagents

REAGENT or RESOURCE	SOURCE	Cat No
**Antibodies**		
anti-CD11b	Biolegend	101207
anti-CD11c	Biolegend	117309
anti-CD-25	Biolegend	101907
anti-CD-3	Biolegend	100203
anti-CD4	Biolegend	100407
anti-CD44	Biolegend	103001
anti-CD62L	Biolegend	104402
anti-CD8	Biolegend	372902
anti-CD80	Biolegend	104705
anti-CD86	Biolegend	105113
anti-FOXP3	Biolegend	126405
anti-CD40	Invitrogen	124609
anti-MHC-II	Biolegend	107605
anti-TNF-α	Biolegend	510801
**Recombinant proteins**		
Recombinant mouse GMCSF	PeproTech	315-03
Recombinant mouse IL-4	PeproTech	214-14
**ELISA Kits**		
Mouse IFN	Biolegend	430804
Mouse IL6	Biolegend	431304
Mouse TGF	R&D Systems	DY1679-05
Mouse TNF	Biolegend	430904

Supplementary Material Methods

1.1. Preparation of Heeraka Bhasma (Ayu_ND)

A herbomineral mixture of Heerak Bhasma (HB) or Ayu_ND was supplied by the Department of Rasashastra, J. B. Roy State Ayurvedic Medical College and Hospital, West Bengal, India. Briefly, the Heerak Bhasma (HB) or Ayu_ND (incinerated diamond powder) was prepared from the raw diamond powder (procured from the Diamond market Surat, Gujrat, India) by following the purification and incineration methods mentioned in the texts of Ayurveda (Supplementary Figure **S1**). The purification treatment of raw diamond powder was performed by heating 180 g raw diamond powder to red hot and then quenching immediately in horse gram () decoction. Regarding the horse gram or kulthi bean decoction, 180 g seeds of *Macrotyloma uniflorum* (Lam.) Verdc [procured from Amazon, India] was soaked in 750 ml distilled water and boiled three times for 2 h and the extract were filtered. The quenching procedure was repeated for twenty-one times. The obtained powder (175 g) was taken in a mortar; levigation was performed with the 10 ml human serum albumin for six hours; and pellets were prepared and dried. These materials were taken in between two earthen saucers and the junction was sealed. The arrangement is called ***Sharava Samputa*** in parlance with Ayurveda. It was placed inside a muffle furnace and heating was performed at 750°C for 1 hour. After self-cooling, the pellets were collected and made into powder form. Levigation by human serum albumin and heat treatment was performed for five times. The obtained powder was further taken in a mortar; levigation was done by horse gram (*Macrotyloma uniflorum* (Lam.) Verdc) decoction for six hours; pellets were prepared and dried. Those were taken in between two earthen saucers and the junction was sealed. The arrangement was placed inside a muffle furnace and heating was performed at 750°C for 1 hr. After self-cooling, the pellets were collected and made into powder form. This time, levigation by decoction and heat treatment were performed for twenty-five times. Finally, 360 g Heerak Bhasma (Ayu_ND) was obtained. The powdered drug was collected and stored in a glass bottle. It was further purified following the Soxhlet extraction process with hexane, dichloromethane and methanol sequentially maintained for 24 h. Finally, the nanoformulations of HB were achieved through distillation and vacuum drying. The end product was highly soluble in a polar solvent and the final pH ranged between 7.2 and 7.4.It was used for various experiments.

1.2. Characterization of Ayu_ND

To determine the phase purity, crystallinity and structure of both the diamond powder (***C_ND***) and *Heerak Bhasma* (***Ayu_ND***) samples, XRD patterns were recorded with an X-ray powder diffractometer (Bruker D8 Advanced, da Vinci) operated at 40 mA and 40 KV with Ni-filtered CuKα radiation. XRD data were recorded within the scattering angle of 20°–80° 2θ with a step size of 0.02° 2θ and scanning time of 2 s/step. FTIR spectra of the ***C_ND*** and ***Ayu_ND*** powders were recorded with an FTIR spectrometer (PERKIN ELMER, FRONTIER) using KBr discs within the 400–4000 cm^{-1} range. The particle size and elemental composition of the ***C_ND*** and ***Ayu_ND*** samples were revealed from the FESEM images and EDS spectrum (Carl Zeiss, Sigma 300) respectively. Microstructure characterization of the ***C_ND*** and ***Ayu_ND*** samples was carried out by analyzing HRTEM (JEOL, JEM 2010) images, which were operated at 200 KV and equipped with a GATAN CCD camera. For HRTEM sample preparation, a pinch of the powdered specimen was dispersed in ethanol and sonicated for a long time. A small drop of the dispersed solution was then placed on a 300-mesh carbon-coated copper grid, dried for overnight and the dried grid was then used for TEM study. The UV-VIS absorbance experiments were carried out in the frequency range of 200–1000 nm using a UV–Vis's spectrophotometer (SHIMADZU, UV-1800, JAPAN). Fourier transform infrared spectrophotometer (FTIR) is the most reliable tool for identifying the types of chemical bonds (functional groups) in the plant extracts. Dried powder of ethanol extract of C. album leaves was used for FTIR analysis. To prepare translucent sample discs, 10 mg of the dried extract powder was encapsulated in 100 mg of KBr pellet by using hydraulic press. The KBr pellet was used as a control. The pellets were loaded in an FTIR spectrometer (Jasco, FT/IR- 4700), with a scan range from 400 to 4500 cm^{-1} in order to determine the functional groups in the ethanolic extract.

1.3. Mice

Female CD-1 [Crl:CD1(ICR)] mice, 6 to 8 weeks of age, were purchased from The Hylasco Bio-Technology Pvt. Ltd, Hyderabad. Animals were housed in pathogen-free conditions of the central animal facility of the department in accordance with the CPSEA guidelines

1.4. Cell lines and generation of DL solid tumors

The murine lymphoma cell lines DL and 2PK3 (purchased from NCC, Pune) used in this study were maintained in complete RPMI-1640 supplemented with 10% FBS. DL is a spontaneous murine lymphoma and was also maintained in the peritoneum with periodic transfer of the tumor

cells to female CD1 mice. DL tumor cells (10^6 cells per mouse) in 100 µL of HBSS were injected into the lower right flank of female CD1 mice. On day 8, when the average tumor volume was 101.5 ± 1.18 mm^3, treatment in mice was started. The tumor volume was measured thrice a week using a caliper [tumor volume (mm^3) = (longer diameter) × (shorter diameter)^2 × 0.4]. Animals were sacrificed when the tumor diameter exceeded 25 mm or when there were signs of animal distress. Survival was recorded as the percentage of surviving animals on a given day.

1.5. Isolation and Characterization of DCs and T-cells from spleen of CD1 mice

Mouse splenic myeloid DCs (CD11c, MHC Class II, CD11b and CD8α) from normal, tumor bearing or disease-free treated mice were isolated as described previously. For the isolation of T cells, single cell suspensions of splenocytes from normal mice, tumor-bearing mice or treated mice were prepared. Purified CD4+ or CD8+ T cells were isolated as described before. Red blood cells (RBCs) were lysed with RBC lysis buffer (Sigma R 7757). Isolated DCs were further incubated with ***Ayu_ND*** (10 µg/mL) or LPS (10 µg/ml) for in vitro stimulation.

1.6. Assessment of in vitro DC mediated cytotoxicity and antitumor activity

DCs were cultured in 96-well plates with medium alone or in the presence of ***Ayu_ND*** (1 mg/mL) or LPS (5 mg/ml) for 16 hours in assay medium. After 1 hour, the cells were washed (×3), and the tumor cells were added to the wells at different E:T ratios depending upon the experimental protocol. The plates were incubated at 37°C, and 5% CO2, for 48 hours. Cell proliferation was measured by an MTT assay kit from Promega, USA according to the manufacturer's protocol and absorbance was measured at 570 nm. The data are presented as the percent of inhibition calculated from the following formula:

$$\text{\% Growth Inhibition} = [1 - \frac{\text{Experimental OD}_{570}}{\text{Target OD}_{570}}] \times 100$$

where Exp. OD is the reading of tumor cells cultured with DCs after various stimulations, Effector OD is the reading of only DCs after various stimulations and Target OD is the corresponding value of tumor cells cultured only in the absence of DCs.

The lytic activity of DC against DL or 2PK3 target cells was measured by a cytotoxicity assay Kit (Promega, USA) which quantitatively measures LDH, a stable cytosolic enzyme released on cell lysis. The percent specific lysis was determined by means of the following formula:

$$\% \text{ Cytotoxicity} = \frac{(\text{Experimental - Effector Spontaneous - Target Spontaneous})}{(\text{Target Maximum - Target Spontaneous})} \times 100$$

1.7. Detection of Apoptosis

Evaluation of apoptotic cell death in DL by activated DCs or by nano diamond formulations against DC was assessed by binding FITC-conjugated Annexin-V as described earlier. Light-scatter characteristics were used to distinguish the tumor cells from DCs. After 18 hours of incubation, the percentages of FITC-conjugated Annexin-V positive cells were analyzed by flow cytometry (CytoFLEX Platform - Beckman Coulter).

1.8. In Vivo Therapeutic Study

The generation of DL solid tumors (day 8 post tumor inoculation) was considered day 0 when the average tumor volume was 151.5 ± 1.18 mm^3. The mice received two cycles, consisting of five oral doses of 50 µg/kg body weight ***Ayu_ND*** along with PEG300 in normal saline at days 01, 03, 05, 09, 07 and again at days 16, 18, 20, 22 and 24 (n=12). The tumor volume was measured three times in a week using caliper. Three (3) mice from each group were sacrificed for the collection of serum, tumor and vital organs for further analysis at day 24 when the treatment schedule was completed. Survival was recorded as the percentage of surviving animals on a given day. Surviving mice had no sign of tumor when experiments were terminated.

1.9. **Secondary tumor challenge**.

To determine the persistence of tumor specific immunity in the mice treated with ***Ayu_ND***, mice showing complete regression of DL solid tumors were given a second subcutaneous tumor challenge (5×10^6 DL) in the left lower flank at day 90 after the first tumor inoculation (contralateral to the first injection site). These mice, as well as the fresh control group of that received the same number of tumor cells (5×10^6) were monitored for tumor size and survival.

1.10. Histopathological analysis

Tumors, liver and spleen from the untreated and treated groups were removed at day 24. Tissue specimens were fixed in 10% neutral buffered formalin overnight, cut into 5 µm thick sections, and stained with hematoxylin/eosin or anti-CD8 antibody as described earlier.

1.11. Antigen-Specific T cell Proliferation

CD4+ or CD8+ T-cells were fractionated from splenocytes by the "Panning" method according to the protocol originally developed by *Wysockiet. et al*. with minor modifications. T cells from healthy control mice, DL tumor-bearing mice or surviving vaccinated mice were used as responder cells against whole tumor lysate pulsed (10 mg/mL) and mitomycin C treated (10 mg/mL) stimulator DCs, derived from similarly treated mice. The responder T cells (5×10^5) were co-cultured with stimulator DCs (5×10^3) and incubated for 120 hours. The MTT assay was used to assess cell proliferation as described above. The proliferative response was expressed as a stimulation index based on the under mentioned following formula

$$\text{Stimulation Index } = \frac{\text{Experimental OD570}}{Control\ OD570}$$

where experimental OD represents the proliferation of responder T cells in the presence of DCs (NDCs, DLDCs or TDCs), stimulated with or without whole tumor cell lysate, and the control OD represents the proliferation of responder T cells only.

1.12. Cytotoxicity Assay of CD8+ T Cells Derived from Treated Mice

The lytic activity of $CD8^+$ T cells against DL or 2PK3 target cells was measured by means of an 18-hour non-radioactive Cytotoxicity assay (Promega, USA), which quantitatively measure LDH, a stable cytosolic enzyme released upon cell lysis as described earlier.

1.13. Ex vivo analysis of DCs and T cells following therapy

DCs derived from normal, tumor-bearing (32 days post tumor transplant) or vaccinated mice were stained with anti-mouse TNF-α antibody and analyzed by FACS as previously described. Surface expression of DC specific markers was assessed by flow cytometry with the use of FITC or PE conjugated anti-mouse antibodies. DC mediated antitumor functions were assessed by cytotoxicity analysis against DL tumor cells. $CD4^+$ and $CD8^+$ T cells were isolated from normal, tumor-bearing or vaccinated mouse splenocytes. The Treg/Th balance was analyzed by flow cytometry with the use of FITC conjugated anti-mouse CD25 and FOXP3 antibodies. Memory $CD8^+$ T cells were analyzed by counterstaining the cells using anti-mouse CD44 and CD62L antibodies. For intracellular staining, cells were treated with IntraPrep permeabilization reagent (Beckman Coulter). The cells were washed and stained with fluorescence conjugated anti-mouse antibodies. After incubation, the cells were washed and analyzed by a CytoFLEX Platform - Beckman Coulter.

1.14. Cytokine ELISA

Serum was collected from each group including the naïve mice and was assayed for the presence of cytokines (TNF-α, IFN-γ & TGF-β) by sandwich enzyme-linked immunosorbent assay (ELISA) using Bio Legend's ELISA Max™ assay kit as per the manufactures protocol. Briefly Purified capture monoclonal antibodies against IFN-γ, TNF-α or TGF-β were coated onto NUNC Maxisorp™ 96 Microwell Plates. Binding of cytokines was quantitated using the appropriate detection antibodies: biotin-conjugated IFN-γ, TNF-α or TGF-β. Streptavidin–horseradish peroxidase (HRPO) conjugate and a substrate solution plus hydrogen peroxide were used to provide color development. The optical density of each sample was read at 450 nm using an ELISA plate reader (MaltiskanGo, Thermo Scientific)

1.15. Statistical analysis

Flow cytometry data were analyzed with the use of FlowJo software (version 10.0.5; Treestar). The mean ± standard deviation (SD) value was calculated for each experimental group ($n = 3$ to 5). Differences between or among the groups were analyzed by ANOVA followed by Holm-Sidak post hoc multiple comparison tests with the use of PRISM statistical analysis software (GraphPad). Kaplan-Meier survival plots were generated with the use of GraphPad Prism software (GraphPad), and statistical significance was analyzed by means of the log-rank (Mantel-Cox) test. A value of $p< 0.05$ was considered statistically significant.

Supplementary Figures

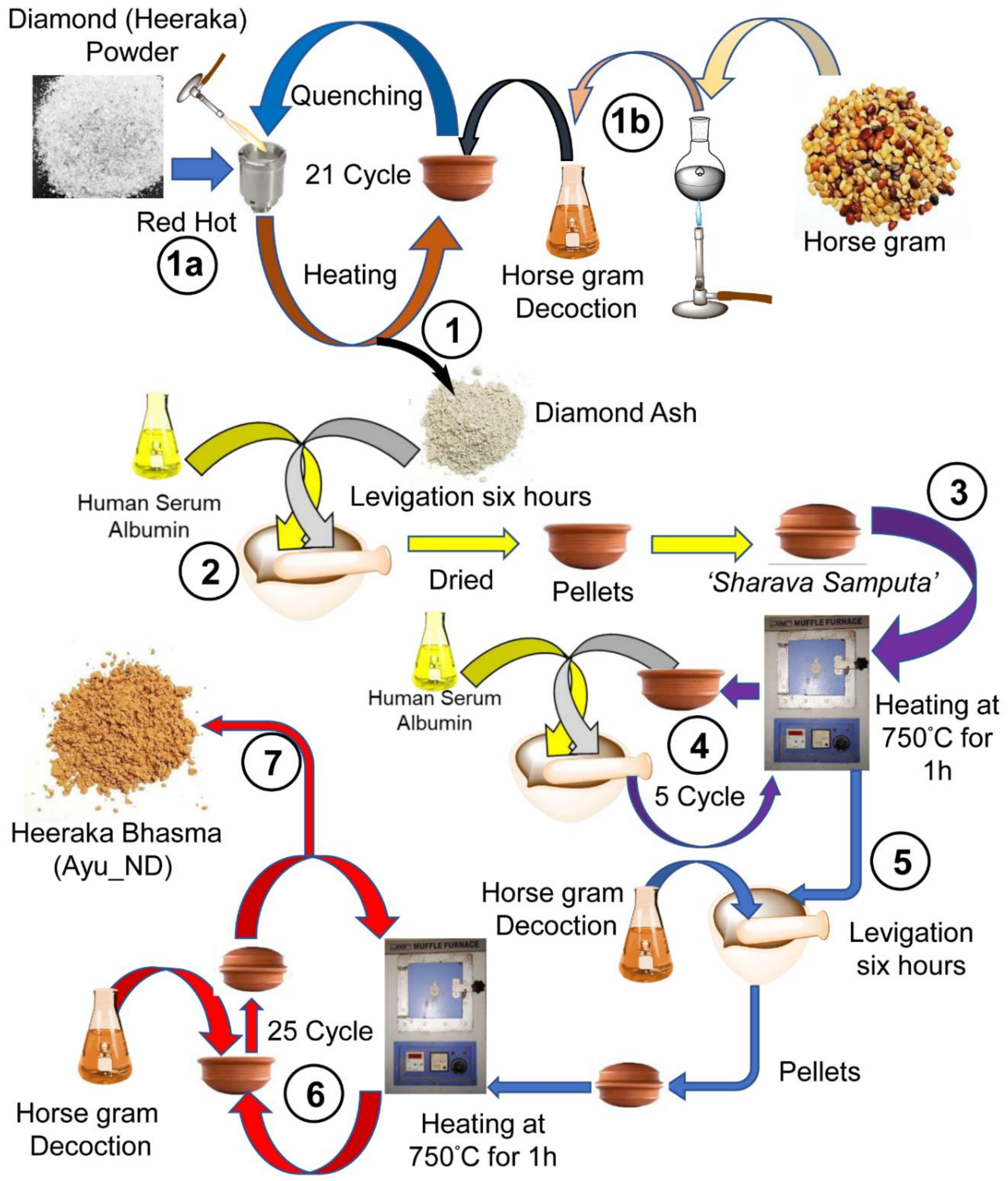

Figure S1 Preparation of Heerak Bhasma (Ayu_ND) *based on the procedure mentioned in the ancient Ayurvedic text Rasaratna Samuccaya.*

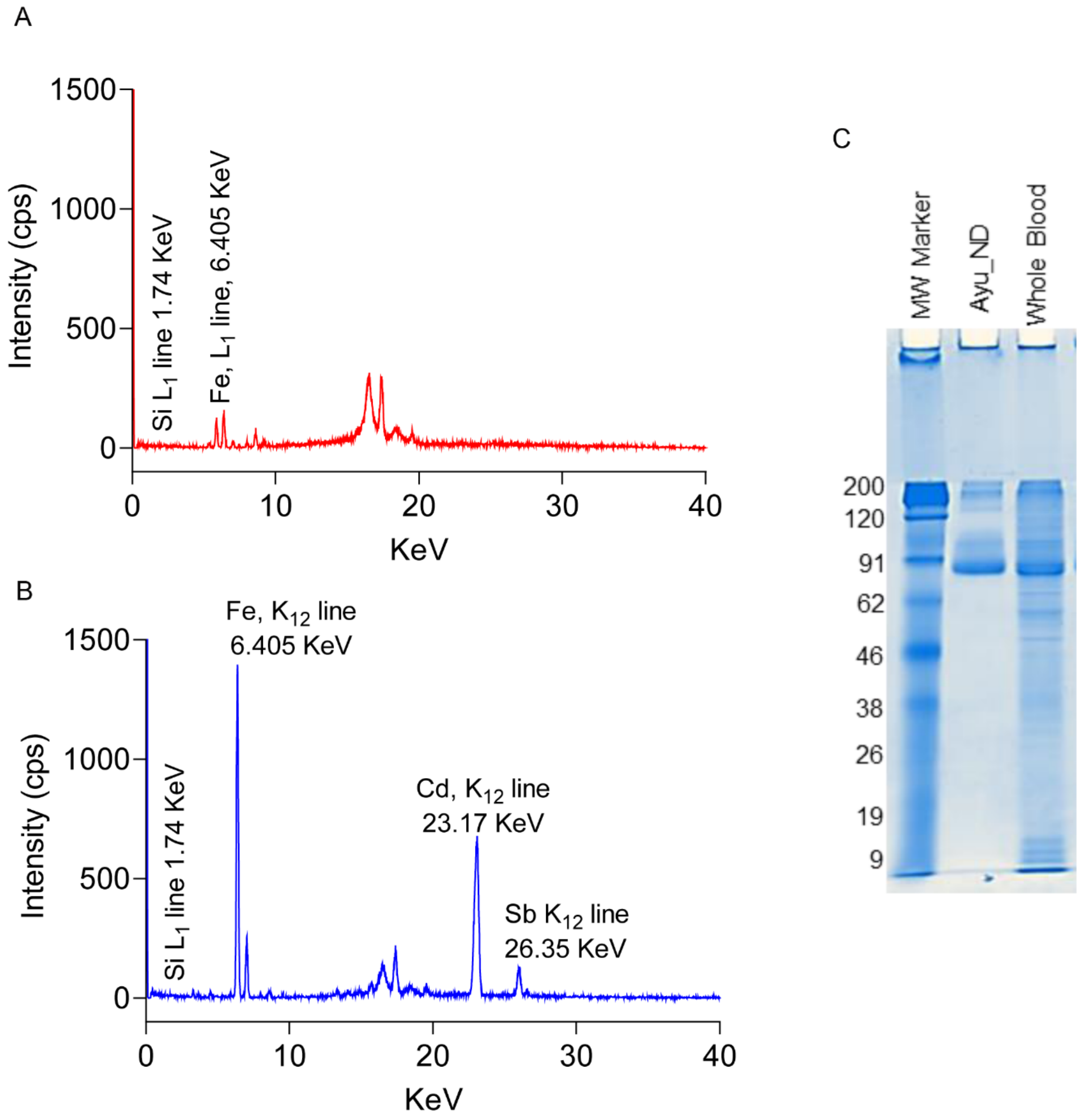

Figure S2 *X-ray fluorescence (XRF) analysis of C_ND (A) & Ayu_ND (B). SDS-PAGE analysis of nanodiamond coupled with BSA. The gels were stained with Coomassie brilliant blue to visualize the proteins (blue band) (B).*

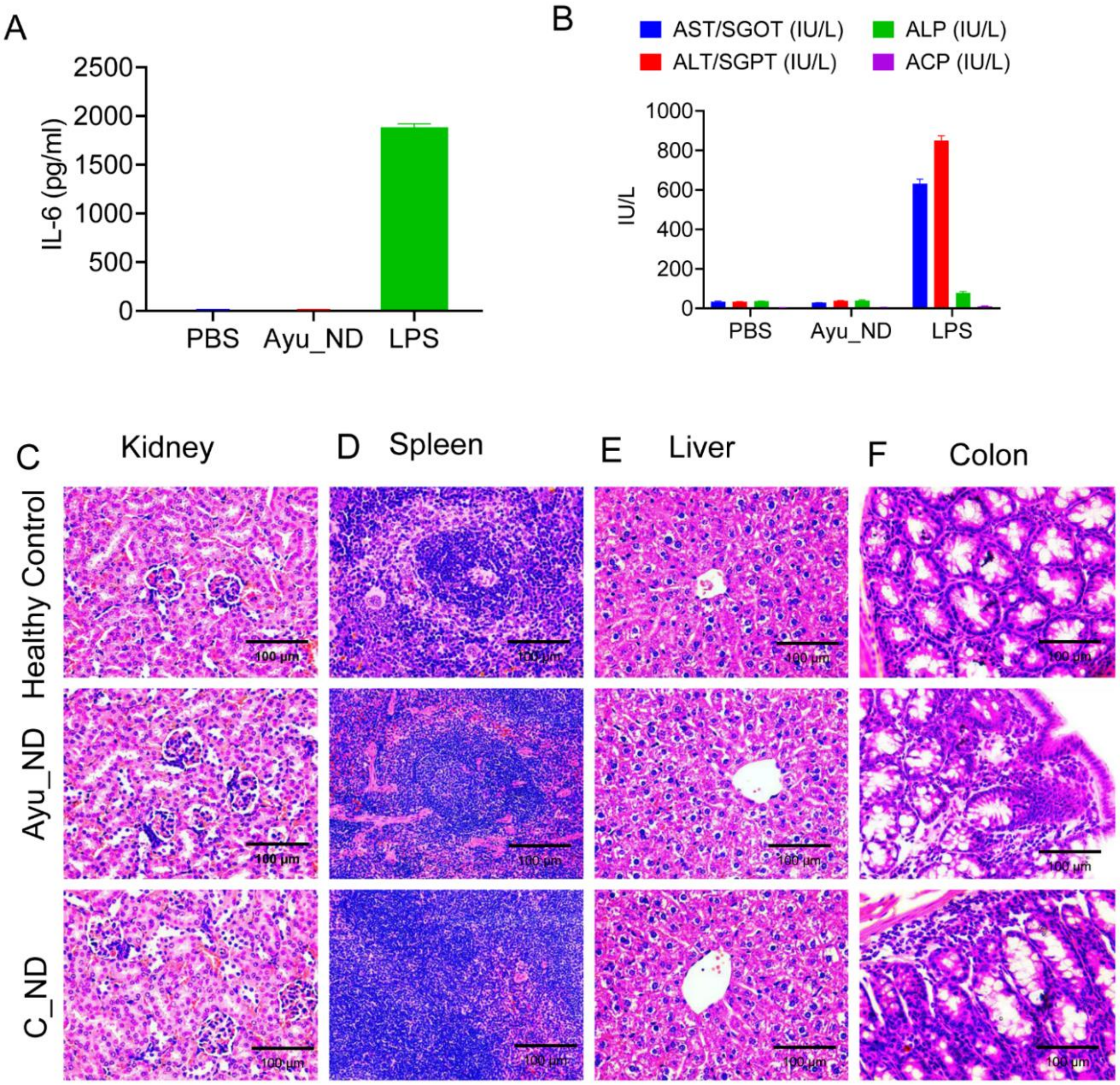

Figure S3. Toxicity analysis of nanodiamonds. *Serum analysis of IL-6 (A), ALT, AST, ACP and ALP (B) was performed in CD1 mice treated with Ayu_NDs or C_ND (200 µg/kg/mouse) (n = 4) or PBS (n = 4) for 1 week or with lipopolysaccharide (LPS) (2.5 µg/kg/mouse) (n = 4) for 6 hours. Data are presented as mean ± SD with triplicate determinations. *P < 0.006; **P < 0.001. Hematoxylin and eosin (H&E) staining for histopathological analysis of of kidney (C), spleen (D), liver (E) & colon (F) derived from the control mice, Ayu_ND and C_ND treated (100 µg/kg of body weight) mice at day 10 post treatment.. Scale bar: 100 µm.*

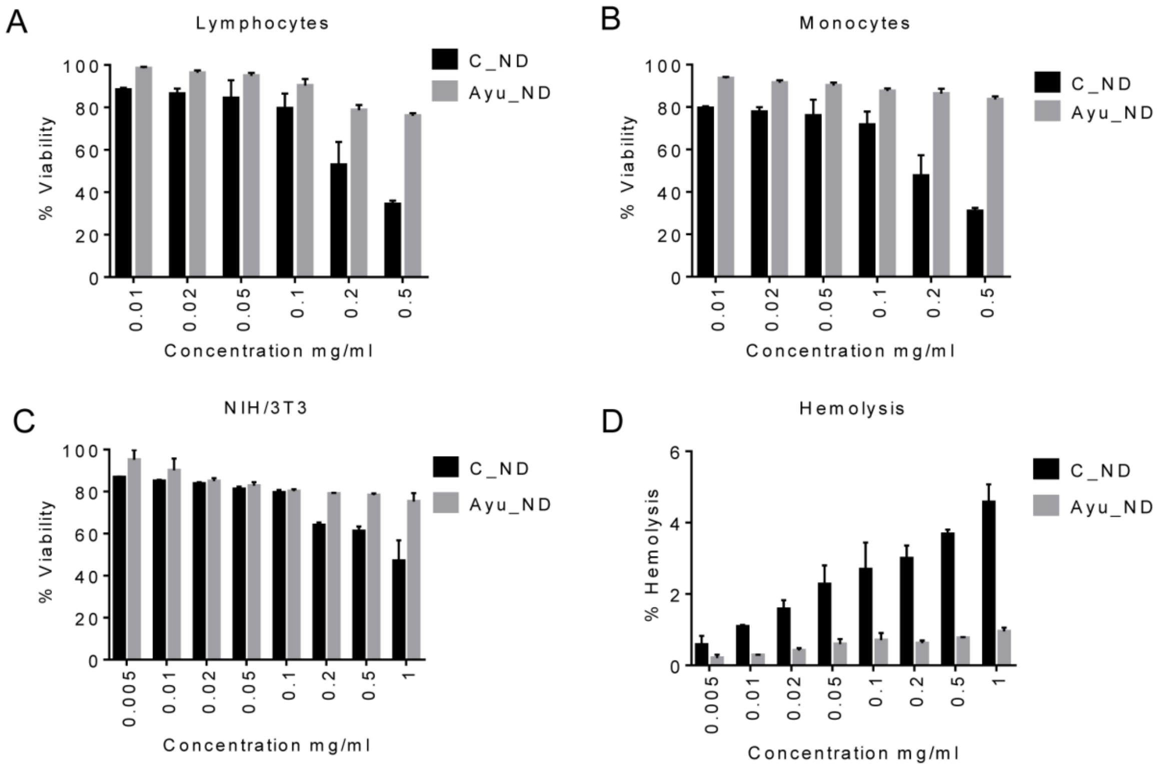

Figure S4 Biocompatibility study of Ayu_ND*. Cell viability of murine lymphocytes (A), monocytes (B), and NIH/3T3 (C) cells after incubation with different concentrations of Ayu_ND or C_ND for 24 h.Hemolysis of red blood cells in the presence of C_ND and Ayu_ND (E).*

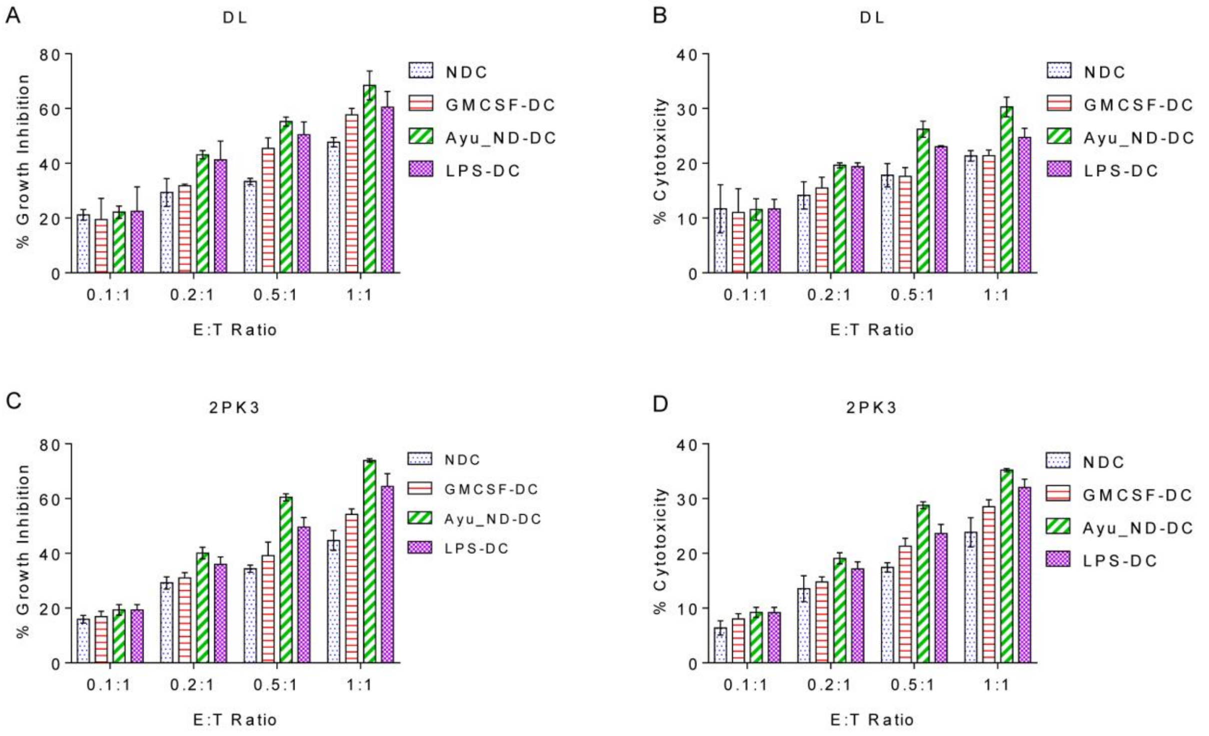

Figure S5 Tumor cell growth inhinitiom and cytotoxicity induced by the nanodiamond formulation. *Naïve (NDC), GMCSF, Ayu_ND or LPS activated DCs derived from the spleen were co-cultured with lymphoma cells (DL) (A) and 2PK3 (C) at different E: T. 48h MTT assay was performed to score growth inhibition by different DCs. A 12h cytotoxicity assay (LDH release assay) was performed using naïve and activated DCs against DL (B) and 2PK3 (D).*

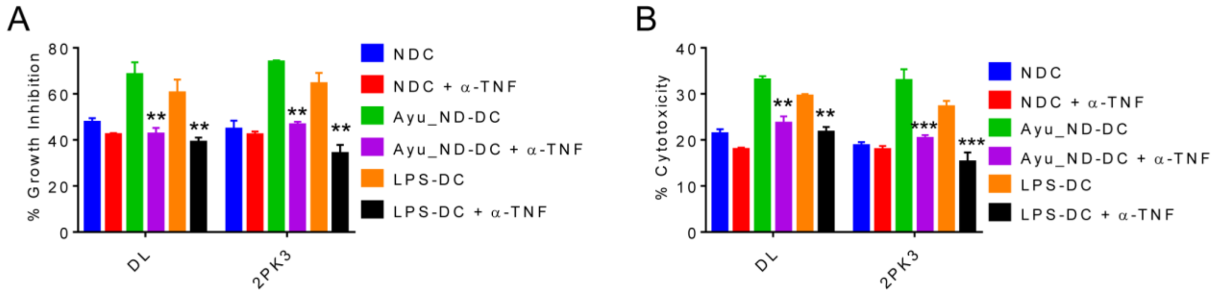

Figure S6 The Ayu_ND activated DC mediated antitumor effect is mediated by DC derived TNF-α*. (A) Splenic DCs from CD1 mice were treated with medium alone, Ayu_ND or LPS for 18 hrs. The cells were washed in complete medium and incubated with or without neutralizing anti-TNF-α antibody for 2 hrs before the addition of DL (n=5, mean ± SD) or 2PK3 (n-5, mean ± SD) and incubated for another 48 hrs before performing the MTT assay to assess growth inhibition. In some experiments (B) DC mediated direct cytotoxicity was measured by an 18h cytotoxicity assay.*

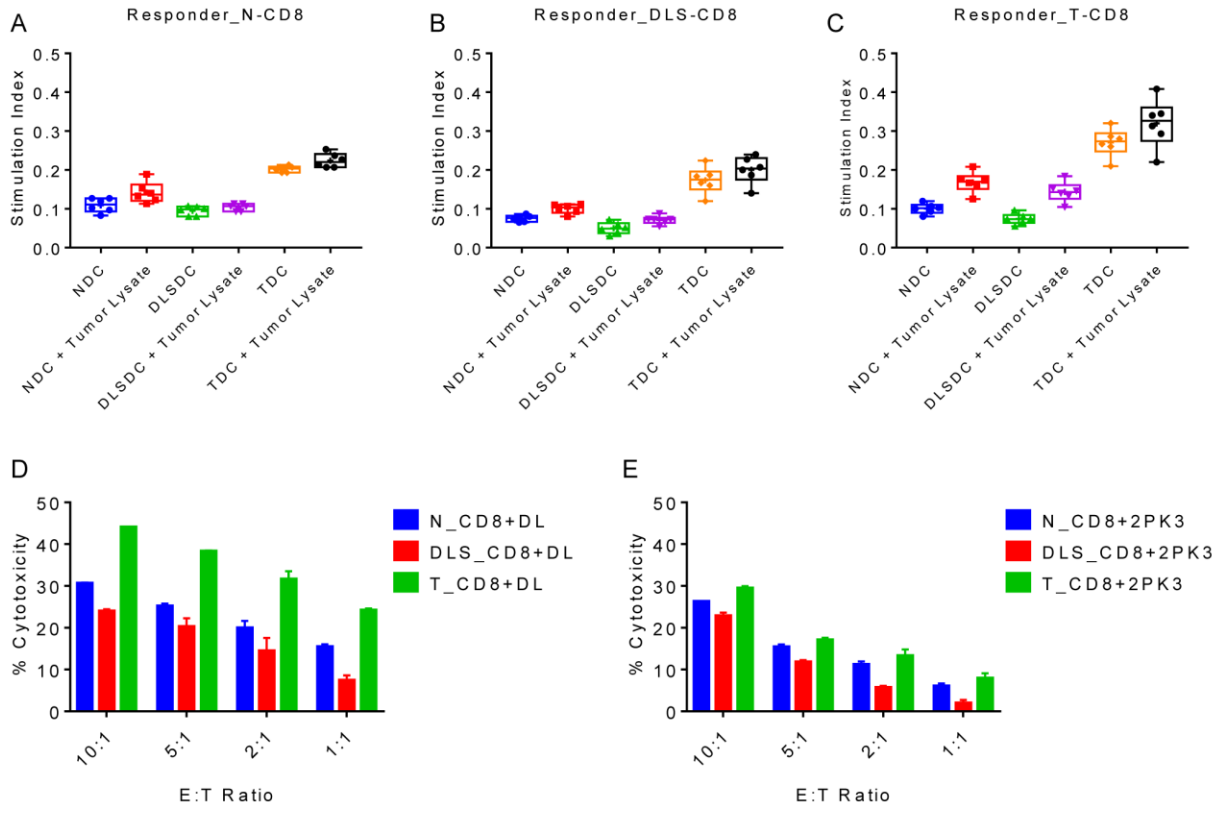

Figure S7 Antigen-specific proliferation and cytotoxicity induced by CTLs. *CD8$^+$ T cells from healthy control mice (A), DL solid tumor bearing mice (B) and tumor free surviving mice following therapy (C) were used as responder cells in the presence of whole tumor lysate and mitomycin C treated DCs (stimulator) derived from the respective groups. Cells were cultured for 120 h, and proliferation was then assessed by means of the MTT assay to evaluate cellular proliferation. The results are presented as antigen-specific proliferation and represented as the ratio of proliferated CD8$^+$ T cells in the absence or presence of tumor lysate pulsed DCs. CTLs from healthy control mice, DL tumor bearing mice and surviving mice were used as effector cells against DL or 2PK3 target cells to assess cell-mediated cytotoxicity (D, E).*

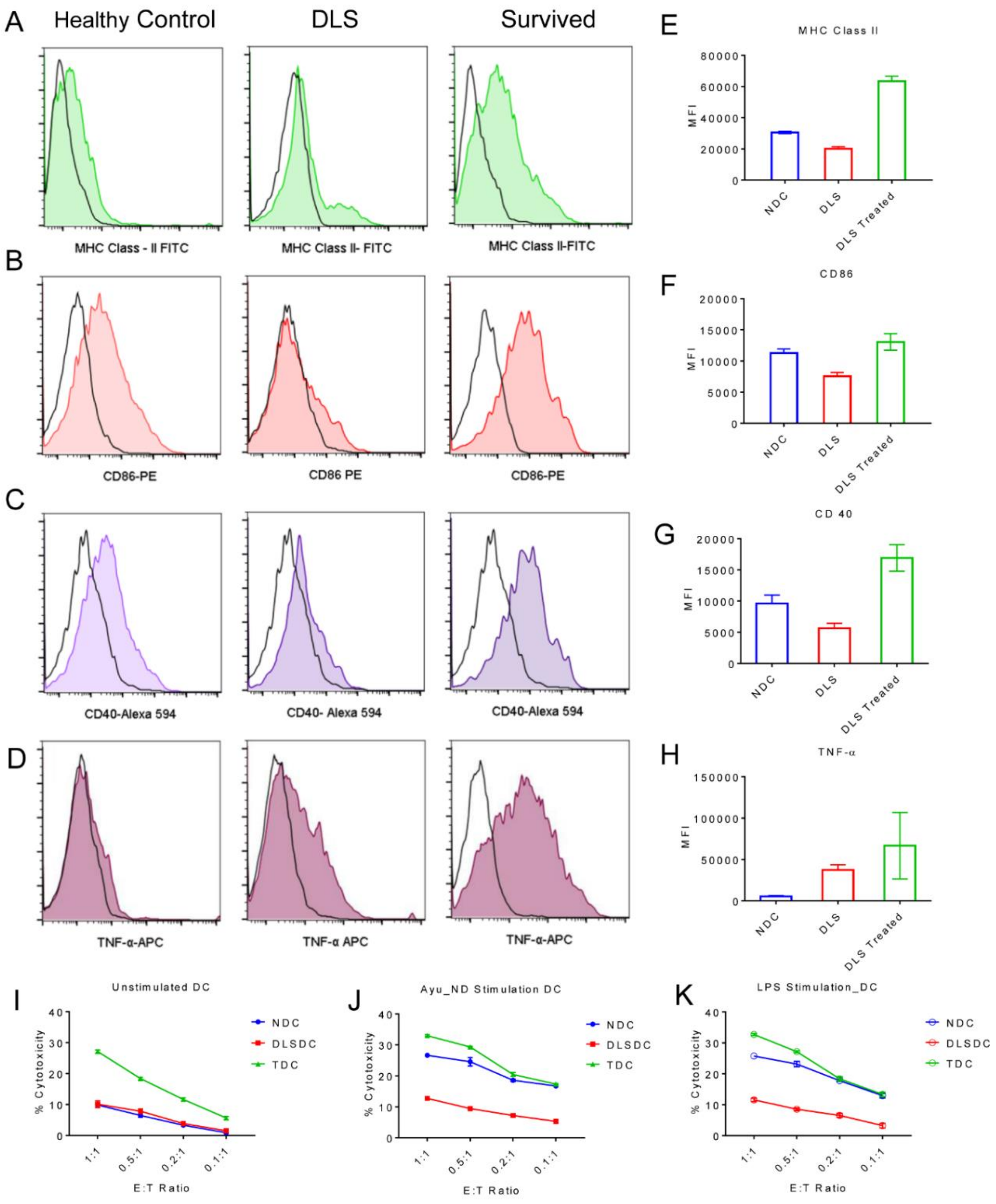

Figure S8 Analysis of effector functions of DCs from Ayu_ND treated animals. *DCs from healthy control, DL mice or DL free surviving mice were isolated and the expression of MHC class II (A) CD86 (B) CD40 (C) and TNF-α (D) was measured by means of FACS. The black uncolored histogram indicates the isotype control. The mean fluorescence intensity (MFI) of MHC II (E), CD40 (F), CD86 (G), and TNF-α (H) expression in dendritic cells in the indicated treatment groups was determined by flow cytometry analysis. Ayu_ND treatment restored the cytotoxicity potential of DCs in DL free treated mice (I-K).*
